# Conformation-Driven Duality in the MBH Proton-Transfer Mechanism: A Dynamic View of Solvent and Entropic Effects

**DOI:** 10.1021/acsphyschemau.6c00040

**Published:** 2026-06-24

**Authors:** Patrick R. Batista, João Pedro B. da Silva, Dario Baum, Barbara Kirchner, Cláudio F. Tormena

**Affiliations:** † Mulliken Center for Theoretical Chemistry, 344102 University of Bonn, Beringstr. 4+6, D-53115 Bonn, Germany; ‡ Institute of Chemistry, 28132 University of Campinas, Monteiro Lobato, 270, Cidade Universitária, 13083-862 Campinas, São Paulo, Brazil; § Department of Chemistry and Pharmaceutical Sciences, 159203 Vrije Universiteit Amsterdam, De Boelelaan 1108, 1081 HZ Amsterdam, The Netherlands

**Keywords:** molecular dynamics, metadynamics, proton transfer, reaction mechanism, free energy, entropic effect, NMR

## Abstract

Understanding how the solvent structure and molecular conformation dictate reaction mechanisms is essential for advancing organocatalysis. In this work, the solvent effect on the proton-transfer step (rate-determining) of the Morita–Baylis–Hillman (MBH) reaction in a protic medium was investigated through a combination of *ab initio* molecular dynamics, well-tempered metadynamics, and on-flow NMR kinetic experiments. The simulations reveal two competing proton-transfer pathways involving solvent-assisted and acid–base mechanisms. These pathways have comparable free energy barriers and are driven mostly by the entropy and conformational preference of the zwitterionic intermediate. Conformational free energy surfaces, combined with NMR analysis, show that bulky substituents in the electrophilic partner may cause a steric effect that hinders proton-transfer accessibility and suppresses MBH adduct formation. These findings establish a direct relationship between conformation and proton-transfer reactivity, highlighting the importance of dynamically addressing the explicit solvation in the MBH reaction.

## Graphical abstract

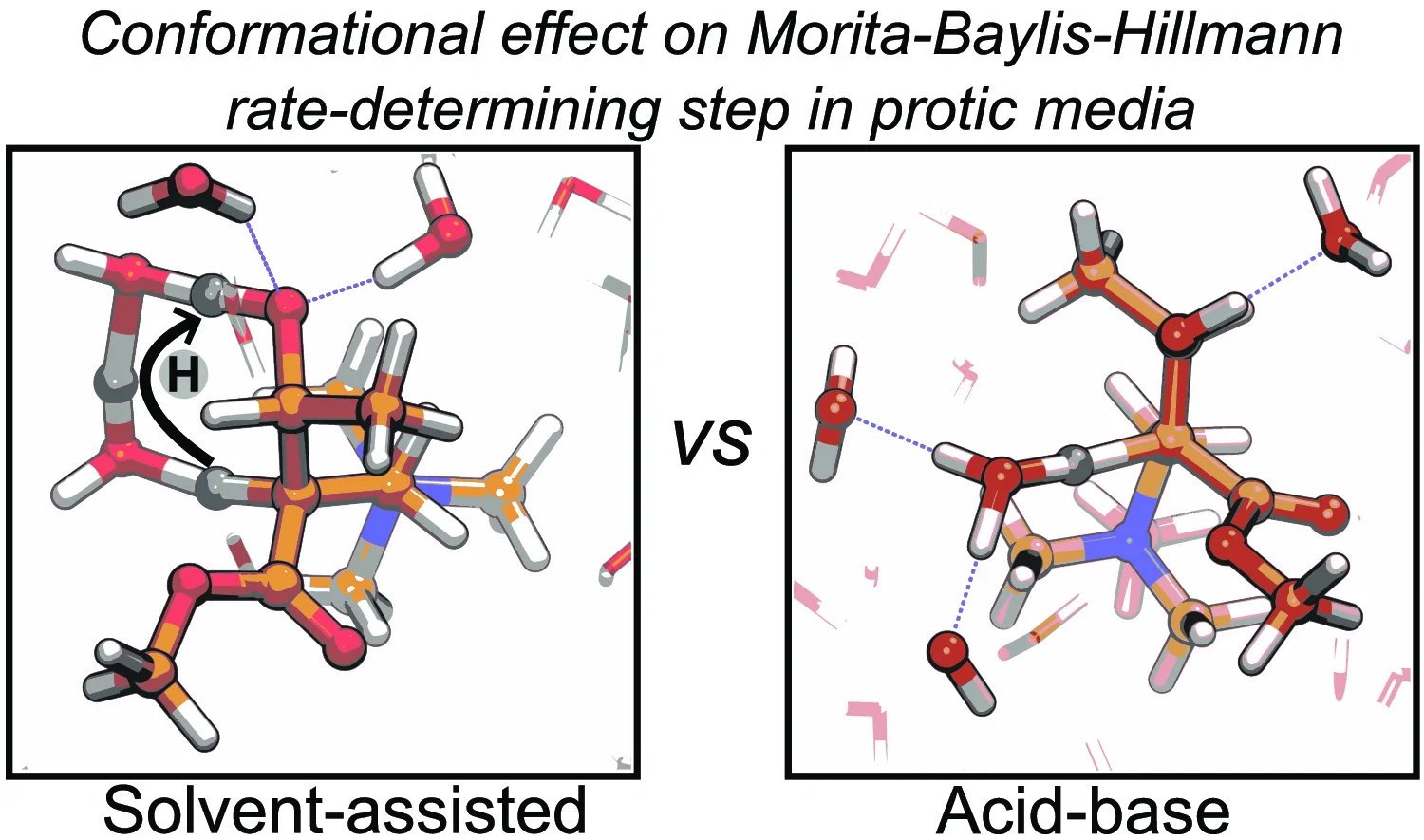

## 1 Introduction

The influence of solvents on chemical reactions has been recognized since the late 19th century.^1^ In 1896,^2^ studies of keto–enol tautomerism of 1,3-dicarbonyl compounds^3^ and nitro-isonitro tautomerism of aliphatic nitro compounds^4^ highlighted the influence of solvents on chemical equilibria. Many remarkable discoveries were made in the following years, including the solvent dependence of UV/vis spectra^5^ in 1878 (later called solvatochromism)^6^ and the use of ionic liquids as a new reaction medium in 1951.^7^ Despite this progress, the effects of solvents have not been given due consideration for many years. This was largely because of the limitations of the available theoretical models at that time, which restricted our understanding of the role of intermolecular solute–solvent and solvent–solvent interactions. Further development has been achieved in the field of solution chemistry in the last few decades,^8,9^ although the understanding of the solvent effect on chemical processes and reaction mechanisms is still challenging and has been intensively investigated.^10,11^

The Morita–Baylis–Hillman (MBH) reaction^12,13^ is an example of a reaction whose mechanism (Scheme 1) is highly dependent on the solvent. MBH is also considered a powerful method for building carbon–carbon bonds,^14,15^ since it uses tertiary amines as catalysts instead of metal-based catalysts, which are typically used in most such reactions. This feature, along with others, allows the MBH reaction to be classified as “green”.^16^

**Scheme 1:**
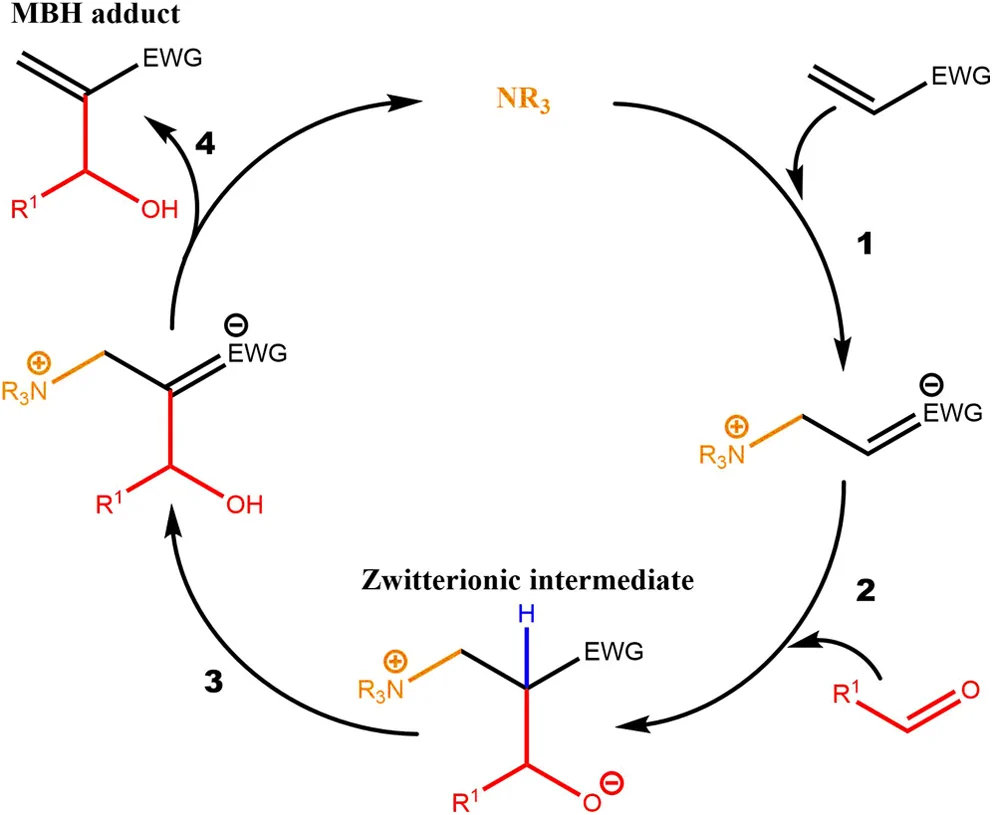
General Representation of the MBH Reaction Mechanism in a Protic Solvent: (**1**) Conjugated Addition of a Tertiary Amine, (**2**) Reaction between the Enolate Formed and an Electrophilic Partner, (**3**) Proton Transfer, and (**4**) Regeneration of the Tertiary Amine Catalyst and MBH Adduct Formation

Different aspects are reported in the literature regarding the MBH mechanism,^17–20^ such as the rate-determining step^21^ and the solvent effect on potential variations in the mechanism and selectivity.^22,23^ Several computational and experimental studies^18,20,24–27^ on the MBH reaction have aimed to accurately compute Gibbs free energies and identify the rate-determining step. Many of them used continuum solvation models, which overlook specific solute–solvent interactions that can affect proton transfer. Other works^28,29^ have adopted hybrid solvation approaches, combining a few explicit solvent molecules with a continuum model. However, this approach still has difficulty accurately reproducing the Gibbs free energy profile.^30^ Based on an experimental study, Plata and Singleton^19^ highlighted some limitations regarding the computational protocols used to investigate the MBH reaction, with respect to free energy calculations, entropic contributions, and the pathway of proton transfer. These limitations led to further investigations employing more accurate computational methods, such as the cluster-continuum model, free energy perturbation, and *ab initio* CCSD­(T).^30^

Despite considerable computational efforts to elucidate the MBH mechanism,^31^ some aspects remain to be further clarified. Therefore, the main goals of this study are as follows: (I) Gain a deep understanding of the protic solvent molecules’ dynamics involved in the reaction process, with a particular focus on the rate-determining step and the nature of proton transfer. Here, we use water to reduce the demand for computational resources. (II) Highlight the effect of zwitterionic intermediate (Scheme 1) conformations and the associated differences in their solvation on the reaction mechanism. (III) Obtain a free energy profile that takes into account these solvent dynamics in the rate-determining step. To address these issues, we applied *ab initio* molecular dynamics (AIMD) along with well-tempered metadynamics (WTMD) simulations,^32–35^ using the reaction shown in Scheme 2 as a model system. This powerful approach allows us to explore free energy surfaces (FES) by selecting collective variables (CVs), which are functions of ionic coordinates (*R*), to characterize the reaction process and distinguish reactants from products.^36–41^ For this investigation, WTMD has a clear advantage over traditional transition state theory (TST) methods, which usually provide a rough estimate of entropic contributions via harmonic frequency calculations. TST also does not capture how solvent molecules change and adapt when they interact with a molecule or transition state (TS) because the interactions with solvent molecules are always considered in equilibrium. On the other hand, metadynamics enables us to include explicit solvent effects, which naturally capture the full entropic contribution at the simulation temperature.

**Scheme 2:**
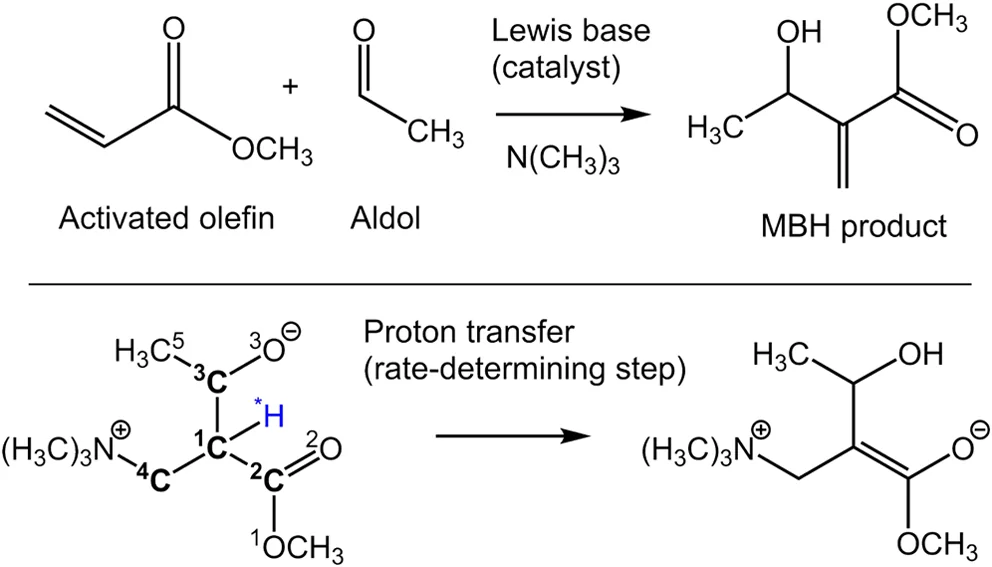
General Representation of the MBH Reaction (Top) and of the Proton Transfer, Considered the Rate-Determining Step (Bottom) Investigated in This Work

## 2 Computational Details

### 2.1 Conformational Search

Since the zwitterionic intermediate may have different conformations with close energies, a conformational search was carried out using a Python code (SUPRA) developed in the Kirchner group. The generation of conformer structures starts after an angle increment of 30° has been chosen. All structures are optimized with the Merck molecular force field (MMFF),^42^ and the duplicate structures are removed. Finally, the final conformations were optimized using DFT calculations at the PBE0/Def2-TZVPPD^43–45^ level of theory, with the continuum solvent model CPCM^46^ for water. Grimme D4 ^47^ energy corrections were used to describe dispersion interactions. These calculations were performed using the ORCA package, version 5.^48^

### 2.2 *Ab Initio* Molecular Dynamics

*Ab initio* molecular dynamics (AIMD)^49^ and well-tempered metadynamics (WTMD)^34^ simulations were performed within the BOMD framework using the Quickstep^50^ module of the CP2K package.^51^ The molecular simulations within density functional theory (DFT) with the revPBE^52^ functional and the hybrid Gaussian basis set DZVP/plane-wave along with the corresponding Goedecker–Teter–Hutter pseudopotentials^53–56^ were carried out to estimate atomic forces for all atoms. Grimme D3BJ^57^ energy corrections were used to describe dispersion interactions. The revPBE+D3BJ method is an excellent compromise between computational cost and accuracy for long AIMD and metadynamics simulations because it provides a reasonable description of liquid water due to partial error cancellation.^58^ Although more accurate, other functionals, such as meta-GGA and hybrid, are computationally expensive, as they require the inclusion of nuclear quantum effects and incorporate a fraction of exact exchange, respectively. The cutoff level of the plane-wave basis set was set at 280 Ry, within the NVT ensemble and with a time step of 0.5 fs.

The three lowest energy conformers from the conformational search were considered as starting points for AIMD simulations. Performing independent simulations starting from these conformers enables the sampling of the dynamical behavior of the system from structurally distinct and thermodynamically relevant regions of the conformational landscape. . The simulated cells were created using the PACKMOL^59,60^ software, consisting of a single optimized conformer of the zwitterionic intermediate in the center surrounded by 64 water molecules with cell dimensions, *L* = 13.18 Å, determined to reproduce the solvent density. Although small, this system size offers a practical balance between computational cost and sampling efficiency. It has been shown to be adequate to remove the most significant problems concerning finite-size effects.^61^ Furthermore, the key interactions that govern proton transfer are predominantly local within the first solvation shell, and finite-size effects are usually less pronounced for structural properties.^61,62^

The simulations were performed in three main steps. First, each system underwent geometry relaxation using Quickstep to eliminate high forces and reach a local minimum on the potential energy surface. Although it does not represent an equilibrated liquid state, this ensures a more stable starting configuration for the AIMD simulations. The configuration was then equilibrated to restore thermal fluctuations and a realistic solvent structure at 400 K using a “massive” thermostat over 5 ps, followed by a 5 ps relaxation step. Finally, a 100 ps production step was performed at 350 K using a “global” thermostat. For water, an elevated temperature alleviates deviations from the experimentally observed structure of liquid water in AIMD simulations.^63–68^ Temperature control during the simulations was maintained using a Nosé–Hoover thermostat.^69^

### 2.3 Proton Transfer and Conformational Distribution in Solution

To investigate the proton-transfer mechanism of the MBH reaction, the final system configuration from the relaxation step was used as the starting point for WTMD simulations. For this, the coordination number (CN) associated with the carbon atom (C1) that loses the proton H* was chosen as CV,^36,70^ as indicated by eq 1. *r*_CH*_i_*_ is the distance between the carbon and hydrogen atoms (χ = |*r*_C1_ – *r*_H*_|), and *r*_0_ is the reference C1–H* distance for bond dissociation equal to 1.6 Å.

1 $${\mathrm{CN}}_{\mathrm{CH}}=\sum_{i=1}^{N_{H}}\frac{1-{\left(\frac{r_{{\mathrm{CH}}_{i}}}{r_{0}}\right)}^{6}}{1-{\left(\frac{r_{{\mathrm{CH}}_{i}}}{r_{0}}\right)}^{18}}$$

To determine the reference distance and expected free energy (*F*) corresponding to the proton dissociation, we performed short AIMD simulations constraining the C1–H* bond distance to a range between 1.09 and 2.09 Å, in increments of 0.2 Å. For each distance interval, we performed a constrained AIMD, from well-equilibrated, unconstrained configurations, to extract the time-averaged forces acting on the collective variable, CV­(χ). Then, a free energy difference can be calculated via thermodynamic integration

2 $$\Delta F=-\int_{1.09}^{2.09}\mathrm{CV}\left(\chi \right)d\chi$$

The simulations were performed over 5 ps, with a temperature of 350 K maintained by means of a Nosé–Hoover thermostat^69^ to obtain reasonable averages of the constraint force. Convergence was verified by tracking the error in the average force for each constrained simulation. Then, using thermodynamic integration, we estimated *F* and the associated distance, which was used to determine *r*_0_ in the CN equation.

The initial height of the Gaussian potential hills, ω, was set as 0.6 kcal mol^–1^, and the Gaussian width, σ, was set as 0.03, which is half of the standard deviation of the C1–H* bond fluctuation observed from unbiased/unconstrained AIMD simulations. In WTMD, we also have to define the parameter Δ*T*, which regulates how quickly the Gaussian height is decreased.^34^ This parameter could be defined from an expected energy barrier of the process by the relation (*T* + Δ*T*) = *XT*, where *X* is the energy barrier in terms of thermal energy, *Xk*_b_ *T*. Using the *F* of the proton dissociation, we set Δ*T* as 23 times the simulation temperature (see also Section S1 for more details and the energy against distance plot). The Gaussian hills were spawned every 50 MD steps, and the simulations were complete after the convergence of the free energy between the two metastable states, with the progress of the deposited bias. Other analyses, such as the evolution of Gaussian height and CV evolution over time, were carried out to ensure the reliability of the WTMD simulations. See the Supporting Information for more details and discussions.

To address the solvent and the structure arrangement effects on the proton-transfer pathway, the electronic structure of a representative TS (RTS) was analyzed using the natural localized molecular orbitals.^71,72^ The orbital analysis was carried out using the NBO6 program,^71^ interfaced with the ORCA program at the PBE0/Def2-TZVPP level of theory, with the RTS configuration obtained from WTMD simulations. For this, we considered a cluster consisting of 32 nearest neighbor solvent molecules around the solute, plus the continuum solvation model CPCM to mimic the bulk effect. The selection of NN solvent molecules was based on the criterion of the minimum distance of interatomic contacts between the solute and the solvent.

It is important to note that solvent coordinates were not explicitly biased in metadynamics because they fluctuate too fast. This makes these variables difficult to control and less effective for distinguishing among different states along the reaction pathway. Thus, they were allowed to evolve naturally within the AIMD framework. Furthermore, to better understand the conformational distribution in solution, we also performed WTMD simulations using the dihedral angles O2–C2–C1–C4 and O3–C3–C1–C4 as CVs (see labels in Scheme 2). The analyses of the production trajectory were performed with the open source program TRAVIS^73,74^ (Trajectory Analyzer and Visualizer: www.travis-analyzer.de).

## 3 Results and Discussion

### 3.1 Conformational Analysis

After performing a conformational search on the geometry of the zwitterionic intermediate by DFT calculations at the PBE0/Def2-TZVPPD/D4/CPCM^water^ level of theory, eight conformers remained as the lowest energies, using an energy window of 5 kcal mol^–1^ and removing the similar structures. These conformers are shown in Figure S2 and labeled as **DFT-C1**–**DFT-C*n*** according to their relative energies. It was observed that the dihedral angles that rotate the methoxycarbonyl (O2–C2–C1–C4) and enolate (O3–C3–C1–C4) groups (see the labels in the 3D structure in Figure 1) are the most important for providing the lowest energy structures. Furthermore, the energy differences are very small, indicating that it is difficult to find the exact conformer that could contribute to the proton-transfer mechanism.

**Figure 1:**
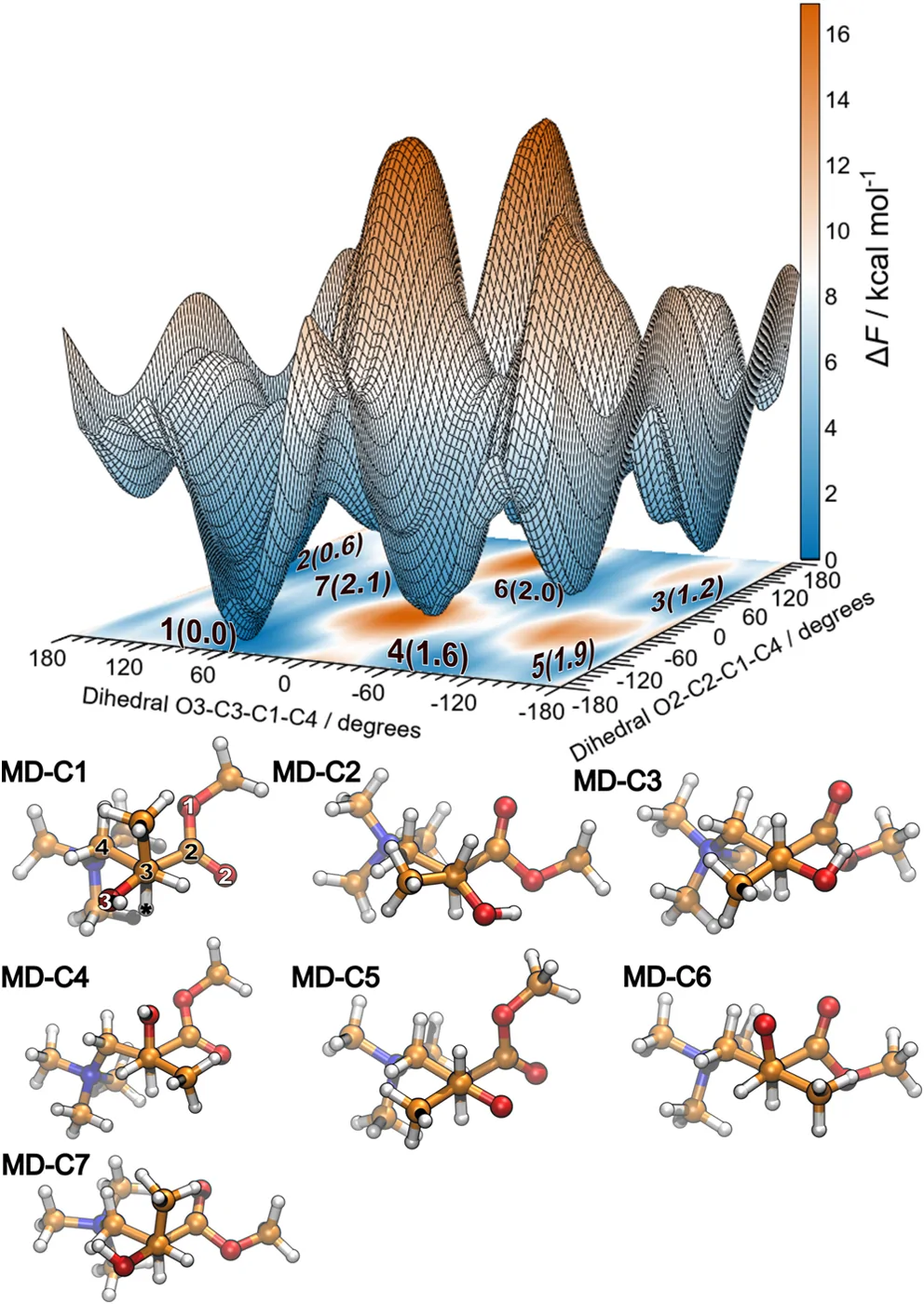
Top: Relative free energy surface from conformational search of the zwitterionic intermediate in water using WTMD with dihedral angles O2–C2–C1–C4 and O3–C3–C1–C4 as collective variables. The inset values represent the relative energies between the found minima. Bottom: Ball and stick representation of the minimum energy conformers. The water molecules were hidden to make visualization easier. The atom colors are H = white; C = orange; N = blue; and O = red.

To investigate the effect of the explicit solvation on the conformational distribution, we performed a WTMD conformational search in solution. The FES, as well as the minimum energy conformers, labeled as **MD-C1**–**MD-C*n*** according to their relative energies, are presented in Figure 1, while the dihedral angles corresponding to each found minimum are summarized in Table S1. The results revealed some differences in comparison with the conformational search using implicit solvation. For example, the lowest energy conformer (**MD-C1**) corresponds to conformer **DFT-C2** in Figure S2, and it is 0.6 kcal mol^–1^ lower than the second minima (**MD-C2**). It is noteworthy that conformer **MD-C7**, which corresponds to the most stable structure from the implicit solvation search, is 2.1 kcal mol^–1^ higher than conformer **MD-C1**. Besides conformer **MD-C1**, the others present even lower energies between them in comparison with those in the implicit solvation. These results suggest that solute–solvent interactions strongly influence conformational distribution and that multiple conformers may contribute to the proton-transfer mechanism. Furthermore, it was observed that most of the lowest energy conformers are protonated on the O3 atom in water solution, which further emphasizes the importance of explicit solvation. Thus, subtle differences can arise from the solute–solvent clusters formed during the TS.

Then, optimized conformers **DFT-C1**, **DFT-C2**, and **DFT-C3** were selected as starting points for the AIMD and WTMD simulations. These structures have the lowest energy, as determined by the DFT calculations, and differ in the orientation of key dihedral angles O2–C2–C1–C4 and O3–C3–C1–C4. Performing independent simulations from these conformers allows for the sampling of the dynamical behavior of the system in structurally and thermodynamically relevant regions of the conformational landscape, thereby avoiding the possibility of the system becoming trapped in a single proton-transfer pathway that could lead to an incorrect mechanistic interpretation.

Plotting the correlation dihedral function (CDF) between the main dihedral angles (Figure 2), it was observed that conformer **DFT-C1** does not change throughout the AIMD simulation, remaining mostly in the same initial conformation. Conformer **DFT-C2** undergoes slightly more changes than conformer **DFT-C1**. The O2–C2–C1–C4 dihedral angle switches from approximately −165° to 180°, with the former angle being the most sampled and corresponding to minimum **MD-C7** on the FES (Figure 1). On the other hand, the O2–C2–C1–C4 dihedral angle changes significantly during the simulation of conformer **DFT-C3**, yielding two main conformations with angles of approximately 150° and 30°, corresponding to **MD-C1** and **MD-C6** minima, respectively. In contrast, the O3–C3–C1–C4 dihedral angle remains nearly unchanged in all three simulations. This behavior is consistent with the FES, which shows that rotation passing by 0° is associated with a high energy barrier. Because of these energetic features, and given that this dihedral is directly associated with the reactive site of the zwitterionic intermediate, three independent WTMD simulations starting from distinct conformers are expected to adequately capture the conformational effects on the proton-transfer mechanism.

**Figure 2:**
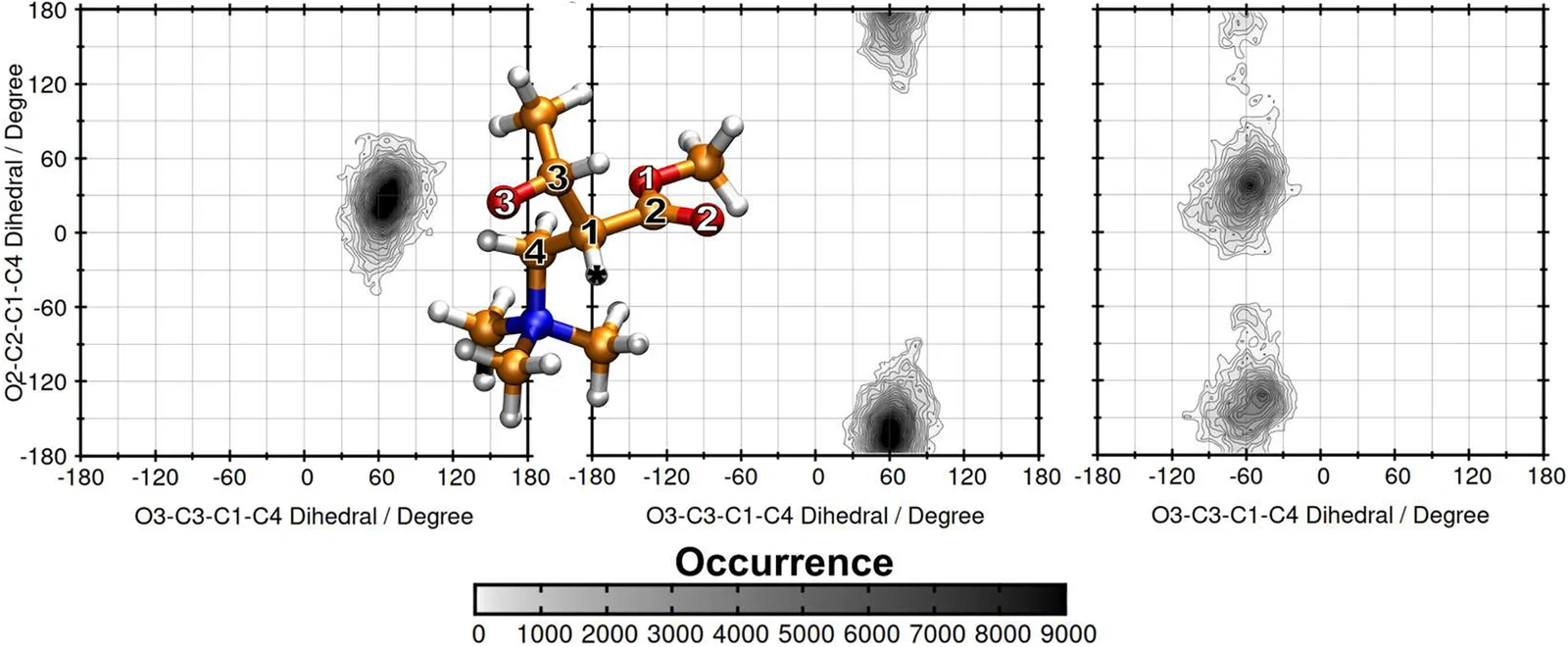
Combined distribution between O2–C2–C1–C4 and O3–C3–C1–C4 dihedral angles during the AIMD simulations for conformers **DFT-C1** (left), **DFT-C2** (middle), and **DFT-C3** (right).

### 3.2 Proton-Transfer Mechanism

To investigate the proton-transfer mechanism, the C–H coordination number associated with the carbon atom (C1) was used as CV, as defined by eq 1. Representative configurations of the transition state (**RTS**) shown in Figure 3 were extracted from the WTMD trajectory and used to analyze the solute–solvent interactions. These configurations are located at the free energy barrier along the selected CV and are based on the CV evolution with time (Figure S7B,D,F). The CV evolution reveals that the C–H bond definitively breaks around 25–26 ps during the simulations of conformers **DFT-C1** and **DFT-C2**, resulting in the deprotonated zwitterionic form. For the simulation of conformer **DFT-C3**, this occurs around 60 ps. It is important to note that a committor analysis^75^ could reveal the TS structure in a more rigorous way, but it was not performed because of the computational cost associated with AIMD. Nevertheless, these representative structures provide consistent mechanistic insights across the different conformers, as will be discussed below.

**Figure 3:**
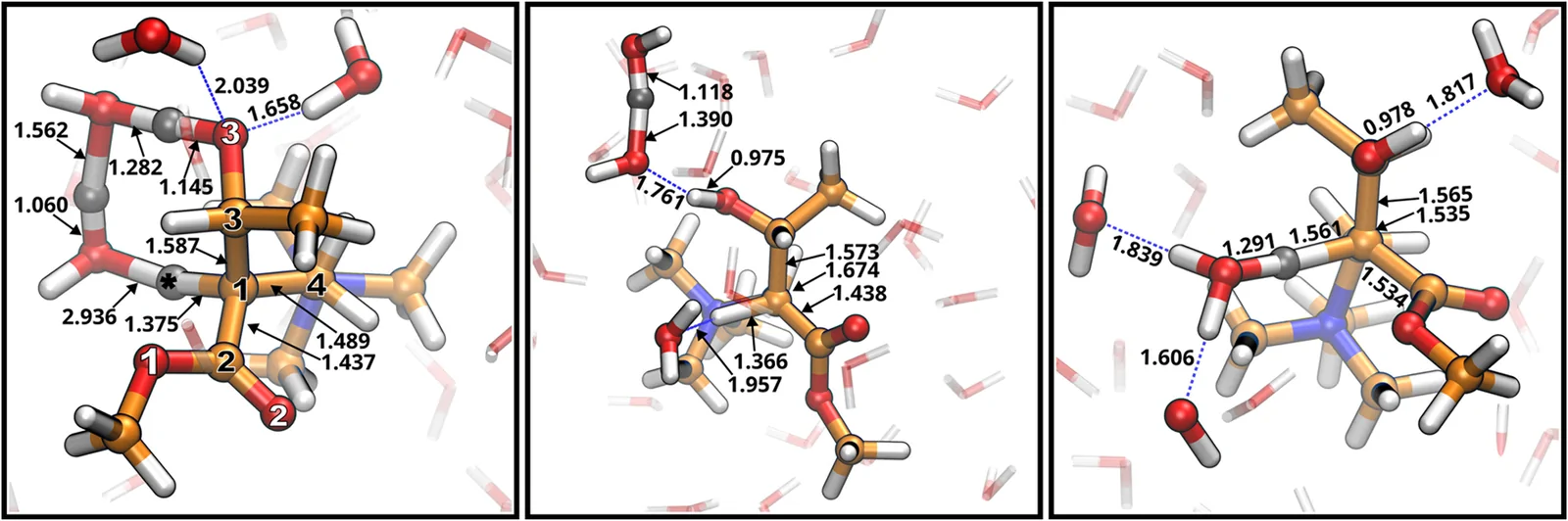
Snapshots from the metadynamics trajectory showing **RTS1** (left), **RTS2** (middle), and **RTS3** (right) representative structures of the proton-transfer step obtained from the WTMD simulation trajectories. The atom colors are H = white; C = orange; N = blue; and O = red. The shared hydrogen atoms between two molecules are highlighted as gray beads. The distance and angle cutoffs of 3.5 Å and 30°, respectively, were considered for counting hydrogen bonds. The main bond distances are shown.

The free energy (*F*) profiles obtained from WTMD simulations are shown in Figure S8A, and the thermodynamic data are summarized in Table 1. The hydrogen dissociation barrier, Δ*F* ^‡^, was found to be 20.0, 20.8, and 23.2 kcal mol^–1^ for the simulation starting with the conformers **DFT-C1**, **DFT-C2**, and **DFT-C3**, respectively. Based on these energy barriers and the RTS structures in Figure 3, it is possible to observe a conformational dependence of the TS, where the O3–C3–C1–C4 dihedral angle plays a significant role. Thus, the angles in **RTS1** and **RTS2**, which present similar energy barriers (*Δ*Δ*F* ^‡^ = 0.8 kcal mol^–1^), are about 60°, while for **RTS3**, which presents the highest Δ*F* ^‡^, it is about 170°. Meanwhile, the dihedral angle O2–C2–C1–C4 has almost the same value in **RTS1** and **RTS3**, ∼40–50°, and in **RTS2**, it is about 90°. However, its rotational barrier is small enough in comparison with O3–C3–C1–C4 (see Figure 1), so that it does not influence the proton dissociation barrier. Additionally, no HB interactions or highly structured solvation was found around the methoxycarbonyl moiety, which could influence the TS structures and consequently their energies.

**Table 1:** Theoretical Thermodynamic Data^a^ (in kcal mol^–1^) Related to the C–H Coordination Number Used as CV: Relative Transition Free Energy (Δ*F* ^‡^), Relative Transition Internal Energy (Δ*U* ^‡^), Relative Transition Entropy Times *T* (−*T*Δ*S* ^‡^); Rate Constant (*k*, in s^–1^) of the MBH Rate-Determining Step Obtained by WTMD Simulations Starting with Conformers **DFT-C1**, **DFT-C2**, and **DFT-C3**

parameter	DFT-C1	DFT-C2	DFT-C3
Δ*F* ^‡^	20.0	20.8	23.2
Δ*U* ^‡^	–1.7	10.3	3.5
–*T*Δ*S* ^‡^	21.7	10.5	19.7
*k*	2.4	0.7	0.02

a Simulation temperature = 350 K.

The different RTSs reflect distinct solvent organizations around the zwitterionic intermediate, leading to specific hydrogen-bonding arrangements that govern the observed proton-transfer pathways. In **RTS1**, an eight-member ring is formed between the solute and two water molecules, where the proton is transferred via the Grotthuss mechanism^76–79^ (also known as a proton shuttle) from the carbon to the oxygen atom (Figure 3, left). Additionally, two more water molecules are observed to stabilize the O3 atom via HB interactions, which also contributes to the proton-hopping mechanism.^80–82^ Conversely, the O3 atom in **RTS2** is initially protonated by the solvent molecules, thereby enabling the newly formed OH group to interact via HB (Figure 3, middle). Concurrently, one water molecule approaches to abstract the proton (H*) from the carbon atom, thus leading to an acid–base mechanism. It is noteworthy that, despite the proton transfer via Grotthuss not being observed in **RTS2**, its solute–solvent arrangement is very similar to that of **RTS1**, but without the proton sharing between two water molecules. This observation indicates that the structure of hydrogen bonding and proton sharing with the O3 atom, likely stabilizing its negative charge, plays a crucial role when O3 and H* atoms are in the same direction. For this reason, the energy barriers for both transition states are almost the same.

The acid–base mechanism is also consistent across **RTS3**. It is characterized by one water molecule approaching the proton, while it is stabilized by two HBs with another water molecule and the OH^–^ ion, as shown in Figure 3, right. Subsequently, the proton transfer to the solvent network occurs via the Grotthuss mechanism. However, the methyl group from the enolate moiety is facing the same direction as the deprotonation reaction. It is noteworthy that the analysis of the trajectories, both visually and through time-dependent RDFs (Figure S5), indicates that the protonation of the O3 atom appears to have a low energy barrier, thereby allowing for multiple proton exchanges with the solvent, even during equilibrium dynamics.^79^ Consequently, the energy barrier for C1–H* bond breaking predominantly governs the MBH rate-determining step in water.

Furthermore, the investigation revealed that intramolecular proton transfer was not observed. The structural arrangement of the four-member ring necessary to enable this process exhibits a higher energy (in the order of 58 kcal mol^–1^) than intermolecular solvent-assisted transfer.^79^ This was also reported by Roy and Sunoj using static calculations considering two explicit water molecules.^28^ Therefore, the intramolecular proton transfer in protic solvents is quite improbable.

In order to deepen our understanding of the factors that affect the proton-transfer mechanism, the free energy was decomposed in terms of internal energy, *U*, and entropy, *S*, components, using the formalism introduced by Gimondi et al.^83^ With this approach, we can obtain a comprehensive overview of the driving energy term and what makes a conformation favorable for the reaction mechanism. Figure S8 shows the free energy decomposition profiles for the three WTMD simulations. On the basis of the results in Table 1, in all the cases, the reaction is exergonic and driven by the entropy component. In the **RTS1** structure, the proton transfer is delocalized over an HB network, which minimizes the energetic cost of proton exchange and results in a negligible or even slightly favorable internal energy contribution at the TS. However, accessing this delocalized proton shuttle configuration requires a specific solvent molecule arrangement, resulting in a substantial entropy reduction that dominates the transition free energy. Although the reaction involving the **DFT-C2** conformer is also dominated by entropy, it presents the largest internal energy barrier with a value that is very close to the entropy contribution. The smaller entropy reduction is characterized by the fewer number of water molecules interacting with C1–H*, thus not stabilizing the proton transfer compared to the simulation of **DFT-C1**. This indicates that **RTS2** undergoes an unfavorable electronic rearrangement, likely due to charge localization and bond stretching, increasing the internal energy contribution. Similar to **RTS1**, proton withdrawal in **RTS3** is stabilized by a structured HB bond network, which leads to a significant reduction in entropy. In contrast, the moderate internal energy contribution indicates that the TS structure is not favorable for proton transfer, resulting in a higher transition free energy. This result can be attributed to steric repulsion between the methyl group attached to the C3 atom and the catalyst moiety.

From Δ*F* ^‡^, the rate constant, *k*, can also be estimated through the well-known Eyring equation: *k* = (*k*_B_ *T*/*h*)­e^(−Δ*F* ^‡^/*RT*)^. Thus, *k* decreases from the simulation of **DFT-C1** to **DFT-C3**, indicating that **RTS1** is predicted to dominate the reaction kinetics under experimental conditions. Further, this result supports the conformational dependence of the reaction step. Despite the absence of experimentally resolved activation energies for the individual steps of the MBH reaction in aqueous medium, the energetic profile obtained in this study shows remarkable agreement with experimental data reported for the same reaction in methanol by Plata and Singleton.^19^ Given the similar hydrogen-bonding capability and proton-transfer characteristics of protic solvents, the experimental results obtained in methanol provide a valuable mechanistic benchmark for evaluating the aqueous phase calculations. In that work, the authors employed kinetic isotope effects to experimentally characterize each elementary step of the MBH mechanism in a protic solvent, providing a detailed energetic description of the catalytic cycle. Their analysis identified the proton-transfer step as the rate-determining process, with an activation free energy (Δ*G* ^‡^) of 21.2 kcal mol^–1^, arising from enthalpic and entropic contributions of −2.3 and 23.5 kcal mol^–1^, respectively. These values are particularly relevant in the present context because they closely match the activation parameters obtained in our calculations. Remarkably, the experimental energy data reported by Plata and Singleton show excellent agreement with the energetic profile associated with **RTS1**, corresponding to the solvent-assisted proton-transfer step identified in this study.

### 3.3 Hydrogen Bond Interactions and Solvent Organization

To obtain a better overview of the hydrogen bond (HB) topology, Sankey diagrams were built, as shown in Figure 4 for AIMD production trajectories. These diagrams consist of showing the HB donors on the left side and HB acceptors on the right side, and from the radial distribution functions (RDFs) calculated for each donor/acceptor pair, the interactions between all of them can be seen. The bar width is proportional to the number of HBs between the two interacting atoms, and the numerical values correspond to the total HB count of the donors or acceptors.

**Figure 4:**
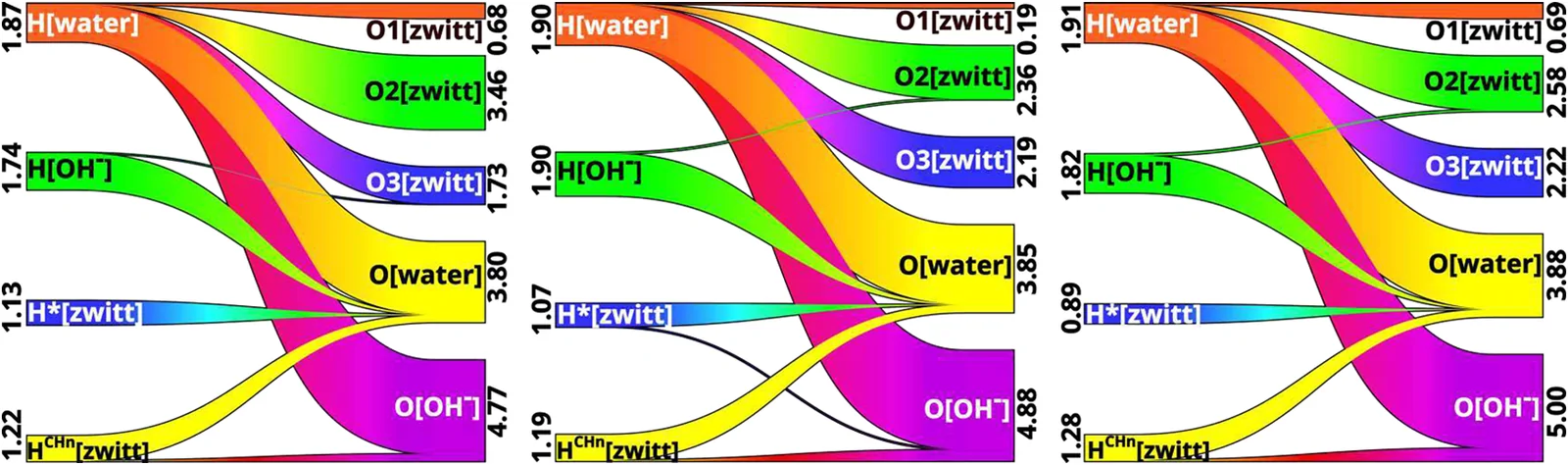
Sankey diagrams showing the hydrogen bond topology during the AIMD simulations starting with conformer **DFT-C1** (left), conformer **DFT-C2** (middle), and conformer **DFT-C3** (right). The groups on the left side correspond to the donor sites, and the groups on the right side correspond to the acceptor sites. The RGB color scale represents both the intensity and distance of the first maximum in corresponding RDF: Red (very large maximum height and very small distance); blue (very minimum height and very small distance); yellow (very large maximum height and very large distance); and white (very minimum height and very large distance). The labels are referred to as (see the atom numbers in Scheme 2): H­[water]: hydrogen atoms from water molecules; H­[OH^–^]: hydrogen atom from hydroxyl ions; H*­[zwitt]: hydrogen atom to be abstracted from the zwitterionic intermediate; H^CHn^[zwitt]: hydrogen atoms from CHn groups of the zwitterionic intermediate; O_1_,O_2_,O_**3**_[zwitt]: oxygen atoms 1, 2, and 3 of the zwitterionic intermediate; O­[water]: oxygen atom from water molecules; and O­[OH^–^]: oxygen atom from hydroxyl ions.

The three Sankey diagrams are very similar, showing the water hydrogen (H­[water]) as the most donor and the hydroxyl oxygen (O­[OH^–^]) as the most HB acceptor in the system. Considering only the zwitterionic molecule, O3 and O2 have similar acceptance for HBs in the simulations from conformers **DFT-C2** and **DFT-C3**. However, O3 presented a slight reduction in the HB number, while O2 presented one more HB in the simulation from conformer **DFT-C1**.

Figure S4 shows that, in WTMD simulations, most of the solute–solvent interactions and HB numbers are similar to those in the equilibrium simulations. However, subtle differences regarding the H*­[zwitt] atom and the OH^–^ ion are observed. H*­[zwitt] presents about one unit more HB, with this increase being a consequence of the higher interaction with O­[water] and O­[OH^–^], indicating that the proton exchange between the solvent molecules surrounding the zwitterionic intermediate increases as the reaction process begins.

The spatial distribution function in Figure S6 also gives us an overview of the solvent molecules and OH^–^ ion organization around the zwitterionic intermediate, depending on the conformer in the WTMD trajectories. In all cases, regions of high water probability indicate a structured solvation shell, while the hydroxyl ions occupy more localized and directional regions associated with reactive and positively charged sites. This arrangement fosters the development of hydrogen bonds, facilitates proton shuttling, and contributes to the stabilization of charge separation along the reaction coordinate.

### 3.4 NLMO Analysis of the Transition State

The electronic structure during the proton-transfer mechanism was addressed by analyzing the localized molecular orbitals (LMOs)^72^ of the RTSs. In this analysis, a set of localized natural bond orbitals (NBOs) that resemble a classical Lewis structure is generated, describing the molecules in terms of localized bonding (fully occupied), nonbonding (unoccupied), and lone pair (LP) orbitals.^84^ Furthermore, the stabilizing interactions between an occupied orbital (donor) and an empty or partially filled orbital (acceptor) based on second-order perturbation theory are also obtained.^85,86^ In addition to the NBOs, the natural localized molecular orbitals (NLMOs) are generated, which have the advantage of incorporating the delocalization effects. When the electronic structure is well-localized, an NLMO is very similar to its parent NBO, resembling a perfect Lewis structure. On the other hand, when the electronic structure is delocalized, the NLMO has a non-Lewis part, with an occupancy of its parent NBO below 2.^72^ Thus, it is possible to obtain a chemically intuitive picture of the electron distribution that is easy to interpret and visualize.^87^

The NLMO isosurfaces, shown in Figure 5, describe the LPs of oxygen atoms (LP_*n*_O) and σC1–H* bonding orbitals. In addition to the orbital visualization, the contributions of each atomic hybrid orbital are summarized in Table S2, including the NLMO delocalization tails. For **RTS1**, the Grotthuss mechanism is illustrated by the isosurfaces shown in Figure 5A-I,A-II. The O–H* bond formation, in Figure 5A-I, is characterized by the LP_2_O from the water strongly delocalized in the region of the C1–H* bond. In turn, the C1–H* bond breaking is characterized by the electron density delocalization of its NLMO mostly toward the carbonyl group (C2–O2 bond), as well as with a minor extension to the amino (C4–N bond) and aldol (C3–O3 bond) groups. A strong HB is indicated by the NLMO assigned as LP_3_O from the water on top. This bond formation is a result of the strong interaction between solvent and solute molecules (as shown and quantified in Figure 6B), leading to high electron delocalization (around 10%) in the O3–H region. In Figure 5A-II, we observe delocalized (5%) water LP_2_O in the O–H region, which corresponds to proton sharing between the two water molecules. Their interaction is quantified in Figure 6A. The isosurfaces in Figure 5A-III,A-IV depict solute–solvent HBs that contribute to the stability of the RTS during the proton-transfer process. LP_*n*_O are delocalized in the O–H region of the water molecules.

**Figure 5:**
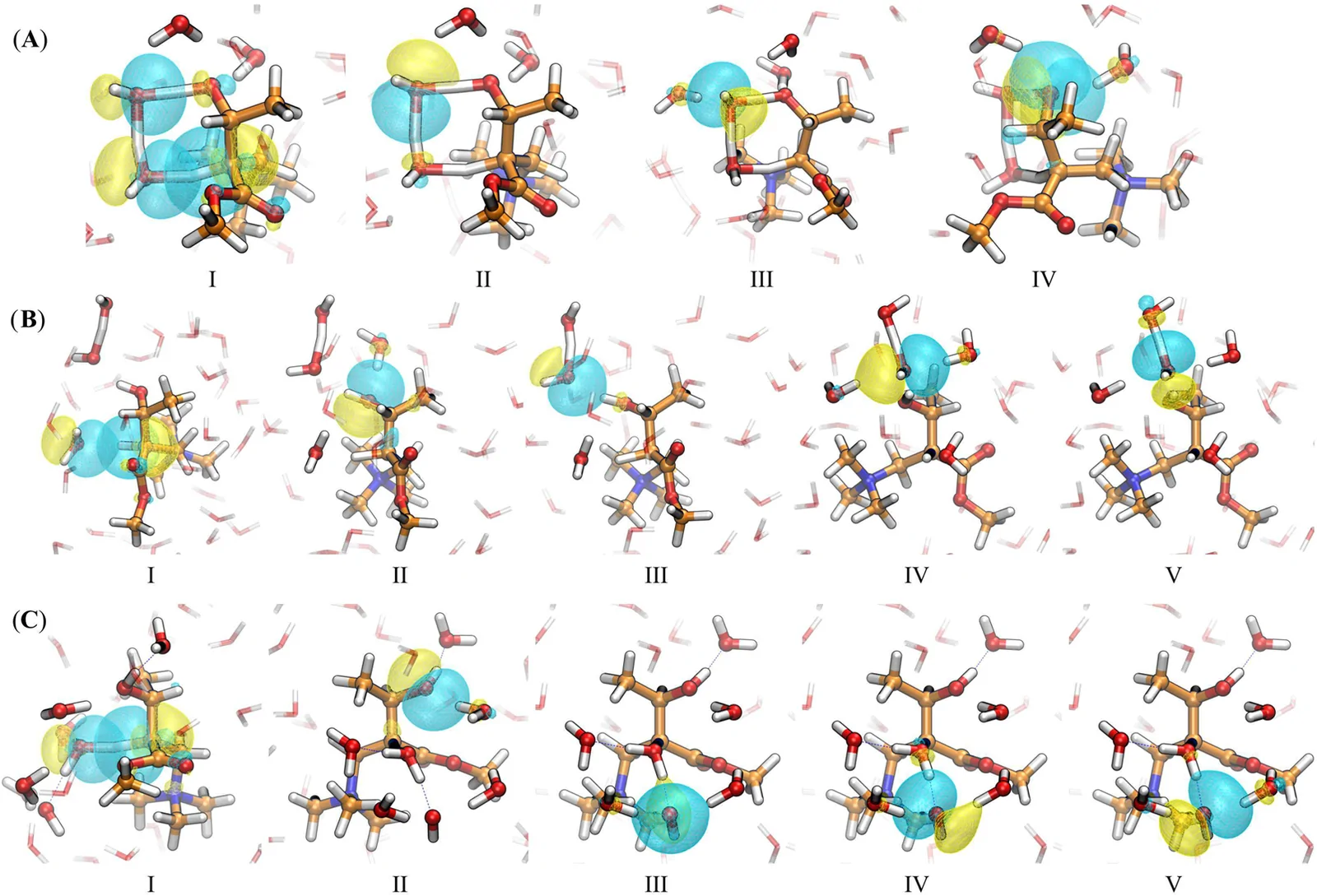
Representations of main NLMO isosurfaces at the proton transfer **RTS1** (A-I to A-IV), **RTS2** (B-I to B-V), and **RTS3** (C-I to C-V). The NLMOs are characterized mostly as the LPs of oxygen atoms and the σC1–H* bonding orbitals. See the NLMO features in terms of s/p/d/f character in Table S2. The atom colors are H = white; C = orange; N = blue; and O = red.

**Figure 6:**
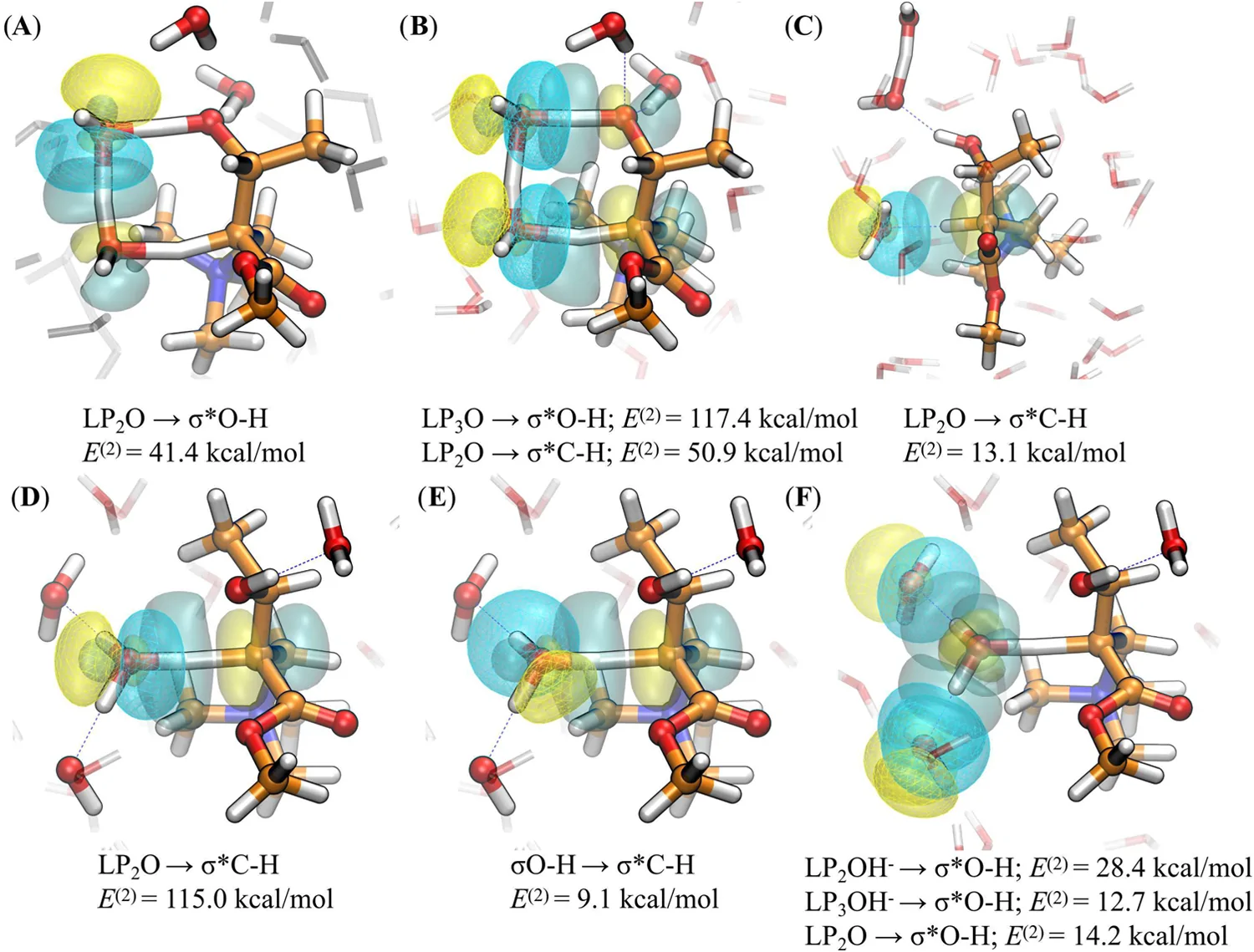
Representation of main NBO isosurfaces of the donor–acceptor interactions and their second-order stabilization energies at the proton transfer **RTS1** (A, B), **RTS2** (C), and **RTS3** (D–F). The wireframed surfaces correspond to the bonding orbitals, while the nonwireframed surfaces correspond to nonbonding orbitals. The atom colors are H = white; C = orange; N = blue; and O = red.

For **RTS2**, the isosurface in Figure 5B-I shows that the LP_2_O from the water molecule is delocalizing toward the C–H* bond. Meanwhile, the electron density of the σC1–H* orbital is mostly delocalized in the carbonyl region. The protonation dynamics of O3 is observed from Figure 5B-III–5B-V. O3–H is stabilized by a cluster formed by the OH^–^ ion and three water molecules. This results in the formation of a hypercoordinated OH^–^ ion that accepts four HB and donates one weaker HB.^88,89^ This clustering features a square-planar arrangement, where the OH^–^ NLMOs assigned as LP_*n*_O have sp (LP_1_O, Figure 5B-III), p (LP_2_O, Figure 5B-IV), and sp^3^ (LP_3_O, Figure 5B-V) type character (see Table S2). Among the three oxygen LPs, sp (LP_1_O) stabilizes the O3–H bond via an HB.

In **RTS3**, the proton-transfer mechanism takes place in a stepwise manner, and it is characterized by an undercoordinated OH^–^ ion accepting three HBs while leaving the donor HB dangling, pointing to a nearby water wire (from Figure 5C-I–C-V). First, the OH^–^ LP_2_O activates proton jumps from the water molecule nearest to H* to OH^–^. Then, H* migrates from C1 to the newly formed OH^–^. Unlike in the previous case, OH^–^ is in a tetrahedral-like arrangement with its oxygen LPs having sp^2^ (LP_1_O, Figure 5C-III), sp^2^ (LP_2_O, Figure 5C-IV), and p (LP_3_O, Figure 5C-V) type character (see Table S2).

Figure 6 shows the main donor–acceptor interactions responsible for the stabilization of the RTS structures. In the three RTSs, the most important interaction occurs between the LP of the water molecule and the C–H nonbonding orbital (LP_2_O → σ*C–H), which characterizes the O–H bond formation and consequently the proton withdrawal from the zwitterionic intermediate. In the acid–base mechanism, the energy of this interaction depends on the approximation position of the solvent molecule, since this may influence the overlap of the LP and σ*C–H orbitals. The second-order perturbation energy of this interaction is *E* ^2^ = 115.0 kcal mol^–1^ in conformer **RTS3** (Figure 6D), with the water molecule being positioned in front of the hydrogen atom. On the other hand, the *E* ^2^ is 13.1 kcal mol^–1^ (Figure 6C) in **RTS2**, where the water molecule is sideways positioned related to the hydrogen atom. This significant energy difference between the two RTS arrangements is due to the stabilization of the interacting water molecule by other solvent molecules via HBs observed in **RTS3**, as shown in Figure 6F. These HBs are identified by the interaction between the oxygen LPs from OH^–^ and H_2_O molecules and the nonbonding σ*O–H orbitals. Additionally, this HB stabilization improves the overlap between the O–H bonding orbital and the C1–H* nonbonding orbital (σO–H → σ*C1–H*), increasing electron delocalization toward the proton and favoring the transfer reaction. Orbital interactions help explain why the proton shuttle mechanism is energetically favorable despite its complex arrangement involving four water molecules and an eight-member ring formation. The RTS involves three strong, high-energy interactions responsible for stabilization and proton migration, as illustrated in Figure 6A,6B. The two water molecules that interact with hydrogen (H*) and oxygen (O3) from the zwitterionic intermediate also interact with each other, sharing a proton. This interaction is characterized by the overlap between the LP orbital of the water molecule on top and the σ*O–H orbital of the water on the bottom, with an energy of 41.4 kcal mol^–1^ (Figure 6A). The other two interactions, as shown in Figure 6B, correspond to carbon deprotonation (LP_2_O → σ*C–H) and oxygen protonation (LP_3_O → σ*O–H) with energies of 50.9 and 117.4 kcal mol^–1^, respectively.

### 3.5 Preferred Reaction Pathway

As discussed above, the conformational preference of the zwitterionic intermediate is largely governed by the O3–C3–C1–C4 dihedral angle, which directly influences the proton-transfer mechanism. According to the energy barriers in Table 1, both the solvent-assisted and acid–base mechanisms are accessible, with the former being energetically favored. However, the solvent-assisted mechanism is only feasible when the molecular conformation aligns the O3 atom with the transferring proton (H*). This spatial arrangement allows solvent molecules to bridge O3 and H*, enabling proton transfer through the hydrogen-bond network.. Based on these results, we propose that the steric effects associated with the alkyl group of the aldehyde (referred to in the following as the **R** group) may influence this arrangement. Thus, when **R** is sufficiently bulky, steric interactions with the catalyst or the carbonyl groups favor rotation around the C3–C1 bond, leading to a conformation in which O3 is oriented opposite H*. In this case, the **R** group faces H*, which may hinder the approach of solvent molecules necessary for proton abstraction. To address this hypothesis, we computed the relaxed free energy surfaces of the zwitterionic intermediate for different **R**-substituent groups derived from pivaldehyde, benzaldehyde, and acetaldehyde. As shown in Figure 7A, for the bulkier isopropyl substituent derived from pivaldehyde, the lowest energy conformer corresponds to an orientation of O3 opposite H* (near −60°), with the **R** group pointing toward the reactive site. In contrast, the conformational minima near 60° for smaller substituents—CH_3_ and phenyl, derived from acetaldehyde and benzaldehyde, respectively—have O3 and H* in the same direction, consistent with a geometry favorable for proton transfer. The steric effect between the **R** group and the amino and carbonyl groups is clearly evident in the conformational maxima, which occur at 0° and 140°, respectively. The maximum for the isopropyl group is almost 3 kcal mol^–1^ higher than those for the methyl and phenyl groups, highlighting the significant steric hindrance. Interestingly, although the phenyl group is larger than the methyl group, both exhibit similar energies at their conformational maxima, as the phenyl group adopts a planar arrangement that reduces steric repulsion.

**Figure 7:**
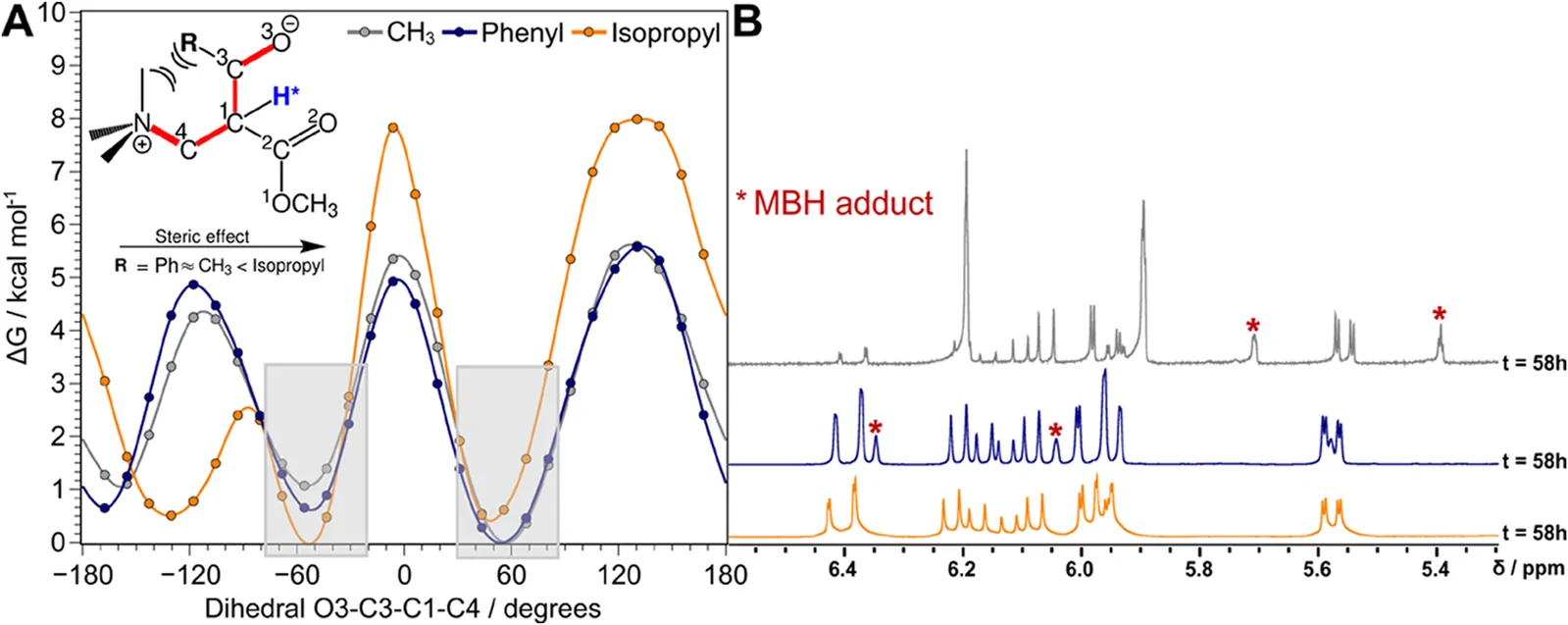
(A) Relaxed free energy surface of the dihedral angle O3–C3–C1–C4 rotation for the zwitterionic intermediate with different aldol—**R**–COH—moieties (**R**: CH_3_, phenyl (Ph), and isopropyl). The points for the lowest energy conformers are in a gray box. (B) ^1^H NMR spectra of the reactions between methyl acrylate and the three different aldehydes (acetaldehyde, benzaldehyde, and pivaldehyde), obtained after 58 h of reaction monitoring.

To further assess this relationship, we used on-flow NMR spectroscopy to monitor the MBH reaction for the same set of aldehydes (see Section S7 for details). This analysis allows for the real-time observation of reactant consumption, intermediates, and product formation. The experiments show that the MBH product was observed for acetaldehyde and benzaldehyde. However, it was not detected for pivaldehyde, even after 58 h (Figure 7B). The full kinetic profiles and the complete characterization of the reaction product and intermediate are also provided in Section S7.

These results highlight the direct relationship between the conformational preference and reactivity in the MBH reaction. Therefore, aldehydes that favor conformers with the protonable site, e.g., O3 facing H*, lead to product formation. Conversely, bulkier substituents shift the conformational equilibrium toward less-favorable geometries, similar to **RTS3**, thereby suppressing or significantly slowing the reaction. This combined experimental and theoretical analysis underscores the role of steric effects in modulating the proton-transfer pathway accessibility.

Despite the presence of certain modeling approaches and significant efforts to assess specifically the rate-determining step, this study successfully identified and addressed the primary challenges of the MBH reaction that have been extensively discussed in the literature.^19,30^ The results also align with the recent work of Kayal et al.,^90^ which highlighted the importance of solvent structural and electronic information in TS structures and catalyst design strategies for tuning the selectivity of organocatalytic processes.

It is noteworthy that the mechanistic insights presented here are derived from free energy profiles obtained using a one-dimensional CV that describes the proton-transfer event. While C–H coordination captures the key chemical transformation and represents a chemically intuitive CV, it cannot entirely rule out the possibility that slow, orthogonal degrees of freedom, including solvent-network rearrangements and conformational changes, contribute to the free energy landscape. Thus, a more explicit treatment of proton transfer, solvent-network rearrangements, and conformational dynamics within a single, multidimensional metadynamics framework could provide a more complete description of the reaction mechanism, in principle. However, identifying a reduced set of collective variables capable of faithfully representing these highly multidimensional processes is challenging. This approach would also increase the computational cost and complexity of the free-energy analysis while introducing potential difficulties in achieving convergence and interpreting the results. Nevertheless, we independently investigated the conformational behavior of the reacting system in solution through metadynamics simulations that employed relevant dihedral angles as CVs. This approach provided a complementary characterization of accessible conformational states. Therefore, the proposed mechanistic interpretation should be considered within the framework of C–H as the CV and the sampling accessible to the current metadynamics simulations.

Furthermore, combining AIMD and WTMD could, in future studies, provide more specific and detailed information about the reaction under different conditions and solvents as well as about other reaction steps.

## 4 Conclusions

The MBH proton-transfer step via acid–base and solvent-assisted pathways were investigated in detail, considering explicit solvation through a combination of AIMD and WTMD simulations. The results provide novel insights into the MBH reaction mechanism in protic media using water as the solvent. The solvent-assisted pathway proceeds through a solute–solvent arrangement involving two water molecules, in which proton transfer occurs stepwise via a Grotthuss-type mechanism. In contrast, the acid–base route involves initial protonation of the oxygen atom of the zwitterionic intermediate, followed by abstraction of the carbon-bound proton either by a solvent molecule or by the OH^–^ anion. Free energy decomposition of the reaction profiles reveals that the acid–base pathway may become energetically less favorable due to steric effects imposed by the aldehyde **R** group. Nevertheless, both pathways lead to exergonic processes, with entropy playing an important role in driving the reaction. Despite the complex TS structure of the solvent-assisted pathway, it is stabilized by solute–solvent and solvent–solvent interactions identified through orbital analysis. Consequently, this route exhibits an energy barrier comparable to that of the acid–base pathway, while yielding enthalpic and entropic contributions in close agreement with experimental data previously reported in the literature.^19^

Although both pathways may occur concurrently, the energy barrier associated with the acid–base route is strongly influenced by the conformation of the zwitterionic intermediate. In this context, the combined conformational and on-flow NMR kinetic analyses demonstrate the subtle effects of the aldehyde **R** group on the conformational preference of the zwitterionic intermediate, thereby affecting the efficiency and outcome of the reaction. Overall, these findings highlight the importance of dynamically evaluating the rate-determining proton-transfer step under explicit solvation conditions to achieve a deeper understanding of the MBH reaction in protic media.

## Supplementary Material



## Data Availability

The XYZ coordinates and trajectories from AIMD and WTMD, NMR spectra files, and free energy decomposition data underlying this study are avaliable at doi.org/10.25824/redu/U5XMOP

## Notes

Details about computational methods, AIMD and WTMD trajectory analysis, NLMO analysis, and on-flow NMR experiments (PDF)
