# Supplementary material for: Conformation-Driven Duality in the MBH Proton-Transfer Mechanism: A Dynamic View of Solvent and Entropic Effects
Source: ACS Phys Chem Au. 2026 Jun 24;6(5):1070–82. doi: 10.1021/acsphyschemau.6c00040 (PMC13614175; doi:10.1021/acsphyschemau.6c00040)
Supplement: Supplementary file 1 [file pg6c00040_si_001.pdf]

SUPPORTING INFORMATION

Conformation-Driven Duality in the MBH Proton Transfer Mechanism: A Dynamic View of Solvent and Entropic Effects

Patrick R. Batista,*[a,b] João Pedro B. da Silva,[a] Dario Baum,[a,c] Barbara Kirchner,[b] Cláudio F. Tormena,*[a]

[a]Mulliken Center for Theoretical Chemistry, University of Bonn, Beringstr. 4+6, D-53115 Bonn, Germany
[b]Institute of Chemistry, University of Campinas, Monteiro Lobato, 270, Cidade Universitária, 13083-862, Campinas, São Paulo, Brazil
[c]Department of Chemistry and Pharmaceutical Sciences, Vrije Universiteit Amsterdam, De Boelelaan 1108, 1081 HZ Amsterdam, The Netherlands.

*email:
patrickr@unicamp.br
tormena@unicamp.br

Contents

S1 Thermodynamic Integration **S3**

S2 Conformational search **S4**

S3 AIMD and WTMD trajectory analysis **S6**
S3.1 Assessment of Solvent S6
S3.2 Sankey Diagrams of Hydrogen Bond Interactions in WTMD simulations S7
S3.3 Time-dependent RDF S8
S3.4 Spatial Distribution Function S10

S4 Assessment of WTMD simulations **S11**

S5 Thermodynamic Analysis of WTMD simulations **S13**

S6 Natural Localized Molecular Orbitals analysis **S14**

S7 On-Flow NMR experiments **S17**

S1 Thermodynamic Integration

Thermodynamic integration yielded the free energy curve shown in Figure S1. The transition state turned out be around $\chi = 1.69$ Å, with a dissociation barrier of approximately 17 kcal mol^{-1}. From these results, the r_0 in Equation 1 in the main text was set as 1.6 Å and the estimated energy barrier in therms of thermal energy ($X \approx 24\ k_bT$) was used to define the ΔT parameter in the well-tempered metadynamics simulations as $(T + \Delta T) = XT$, where T is the simulation temperature. Thus, the ΔT was set as 8050, *i.e.*, 23 times T.

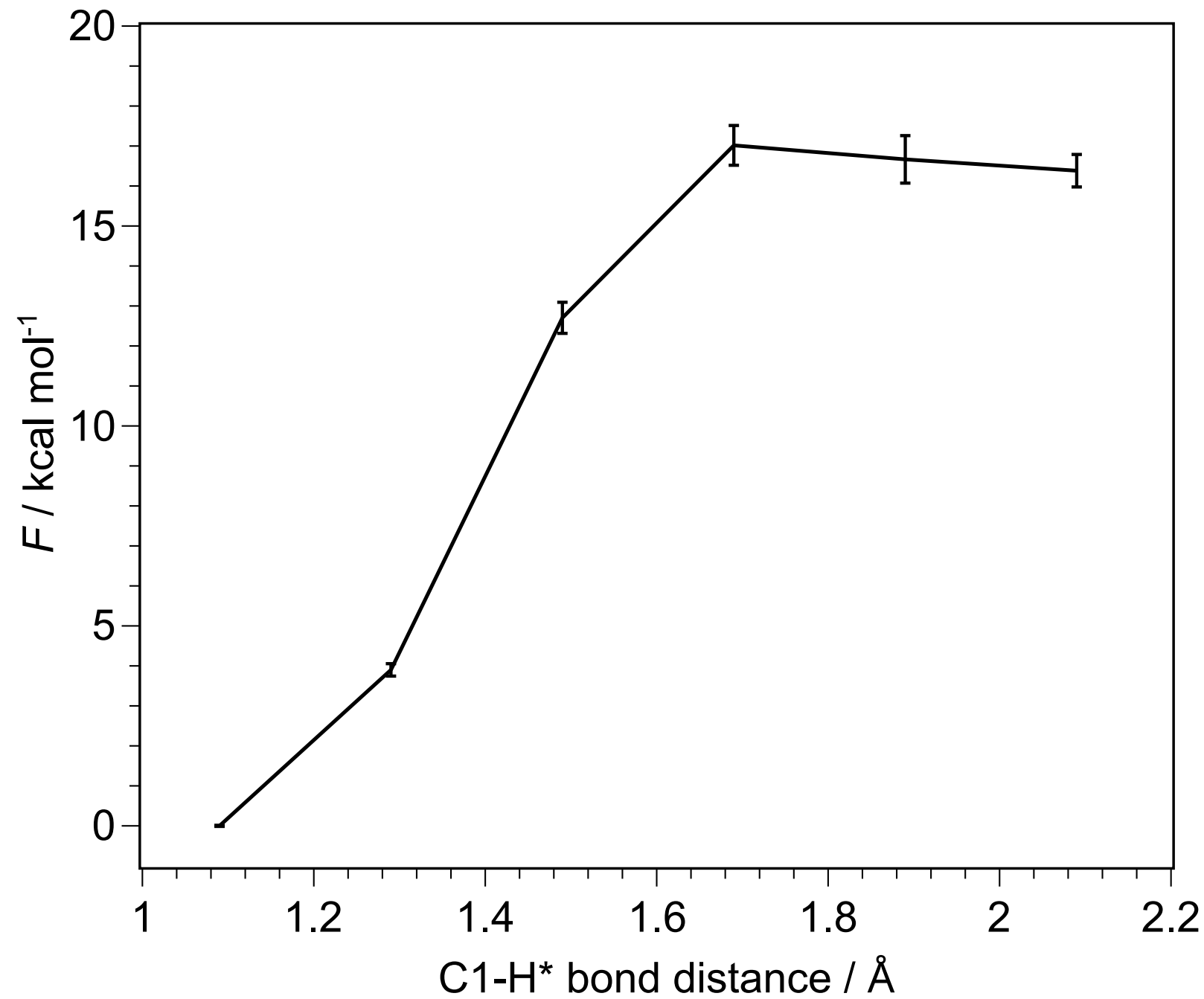

Figure S1: Free energy curve as a function of the constrained C1-H* bond distance for water solvated MBH zwitterionic intermediate.

S2 Conformational search

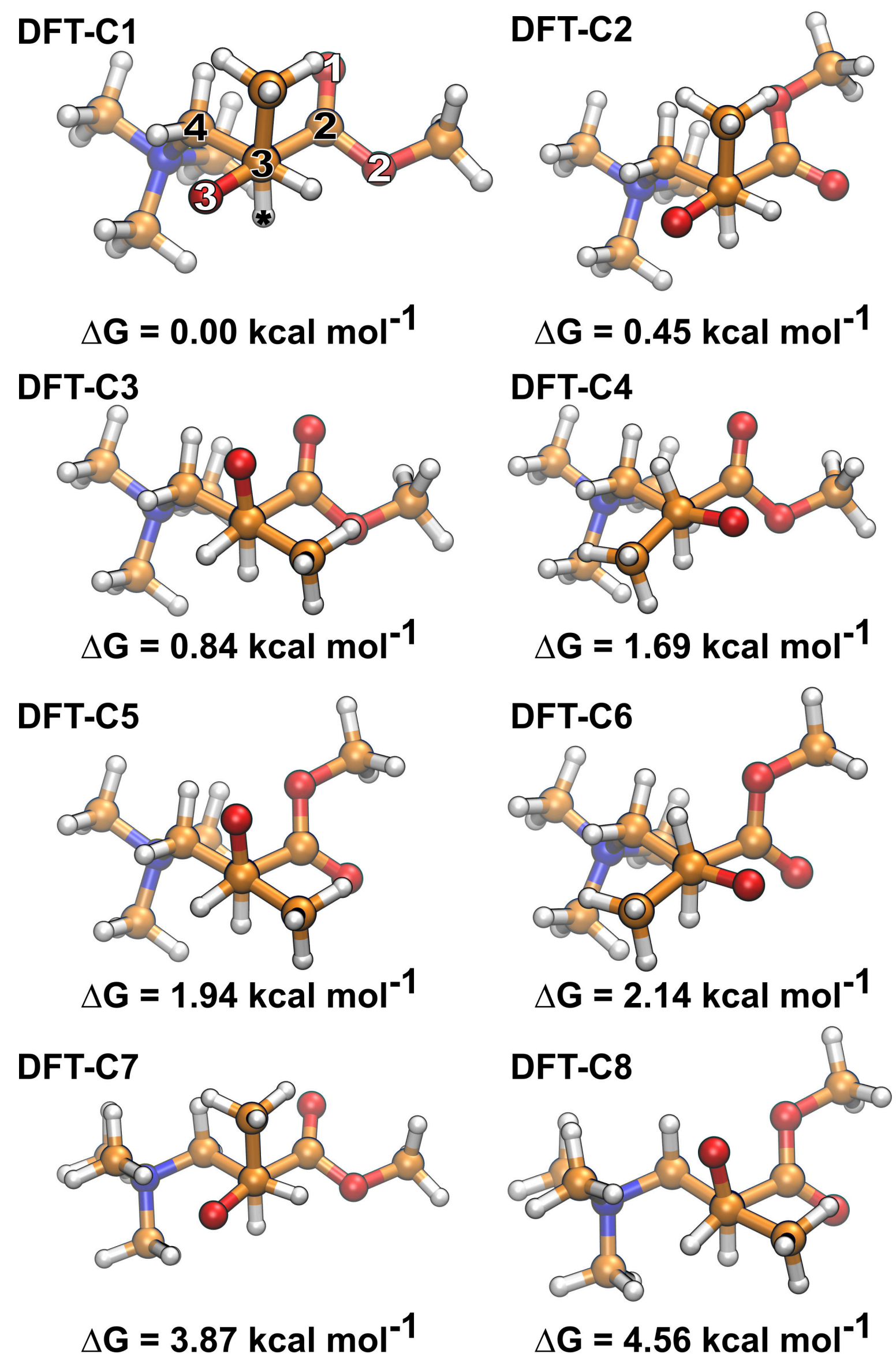

Figure S2: Ball and stick representation of the minimum energy conformers of the zwitterionic intermediate calculated at the PBE0/Def2-TZVPPD/D4/(CPCM^{water}) level of theory. The energies are relative to the global minima conformer **DFT-C1**. The atom colors are: H = white; C = orange; N = blue; O = red.

Conformer	Angle O2-C2-C1-C4	Angle O3-C3-C1-C4	ΔF
MD-C1	-148	66	0.0
MD-C2	37	155	0.6
MD-C3	63	-176	1.2
MD-C4	-153	-73	1.7
MD-C5	-127	180	1.9
MD-C6	56	-56	2.0
MD-C7	20	56	2.2

Table S1: Relative free energies, ΔF, (in kcal mol^{-1}) between lowest in energy conformers of the zwitterionic intermediate obtained from metadynamics in water solution using the dihedral angles (in degree) O2-C2-C1-C4 and O3-C3-C1-C4 as collective variables. See conformer label in Figure 1 of the main text.

S3 AIMD and WTMD trajectory analysis

S3.1 Assessment of Solvent

It is well known that the pure functionals, such as the revPBE used in this work, overestimate the hydrogen bond energy,[1] causing a 'glassy' character of water. This effect is manifested as a higher O-O first peak and a deeper first trough in the oxygen-oxygen (g(r)OO) RDF when compared to RDFs obtained from neutron scattering and/or X-ray diffraction experiments.[2–4] However, the simulations performed at 350 K with Grimme's dispersion correction (D3BJ)[5] minimize such an overstructuring effect. The increase in the simulation temperature also mimic the nuclear quantum effect in structural quantities such as the g(r)OO RDF, which is known to be essential for accurate description of microscopic structure of liquid water.[6] The solvent structure in the simulation was analyzed from the g(r)OO RDFs as given in Figure S3 and compared with the experimental (full gray line) RDF of pure liquid water.

The first set of peaks from 2.45 Å to 5.50 Å presents a small shift and overstructuring in comparison with the experimental data, (inset zoom in Figure S3) which is caused in part due to the presence of the zwitterionic intermediate, reducing the solvent mobility because of the solute-solvent interactions. However, based on the experimental uncertainties,[2–4,7,8] the simulated g(r)OO reflect a reasonably accurate simulation of the intermolecular structure of liquid water.[9]

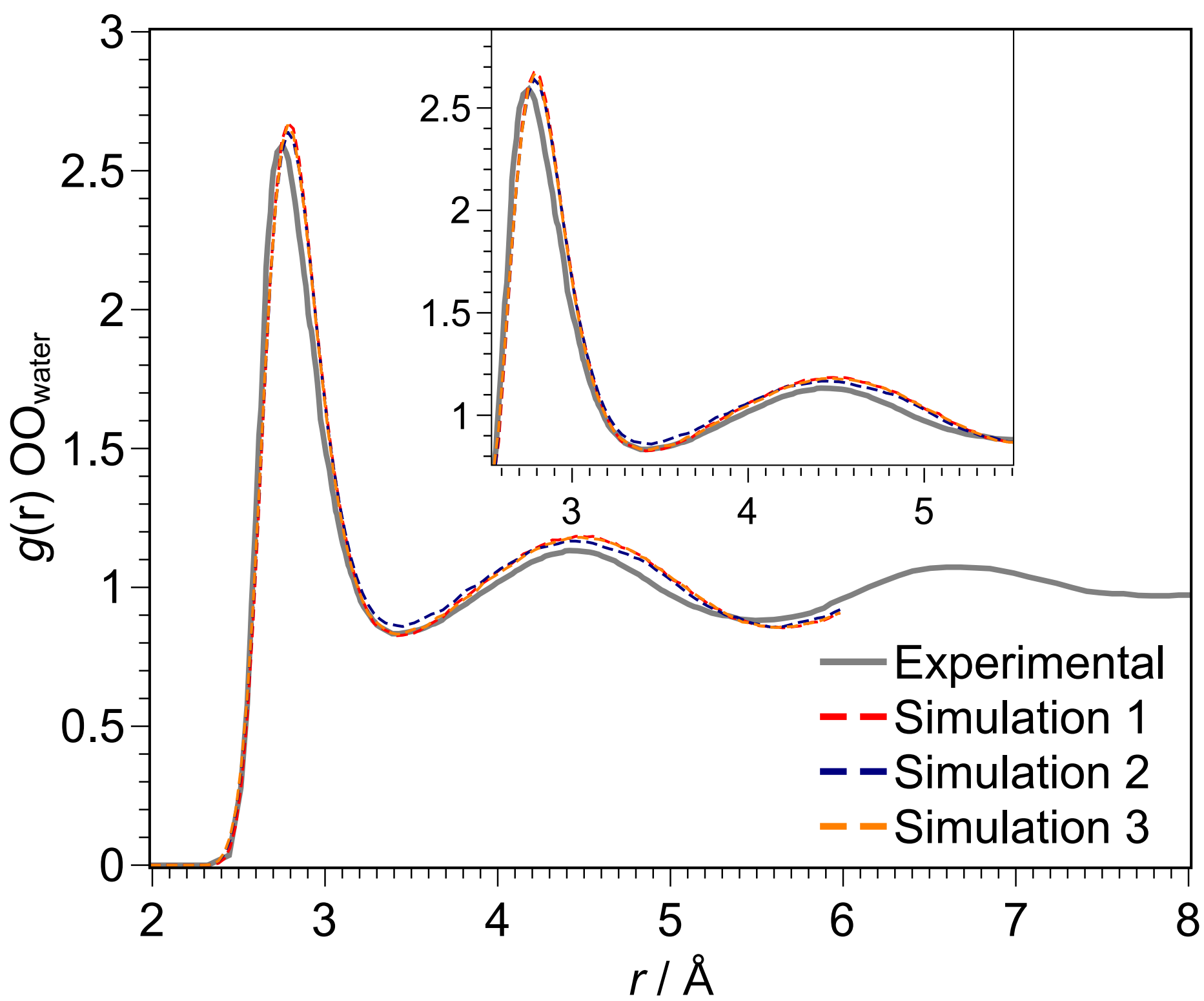

Figure S3: Oxygen-oxygen RDFs for each of the three AIMD simulations of zwitterionic intermediate in water; $g(r)$OO denotes the RDF or pair correlation as a function of pair distance (r) in Å. The full gray line is the experimental oxygen-oxygen RDF for light water from the joint X-ray diffraction and neutron scattering experiments of Soper and Benmore[8]. This latter was extracted using the engauge plot digitizer.[10]

S3.2 Sankey Diagrams of Hydrogen Bond Interactions in WTMD simulations

To address the HB network during the WTMD simulations for probing the proton transfer reaction, an analysis was performed using Sankey diagrams built by the TRAVIS program,[11,12] and shown in Figure S4 (see more details in the main text).

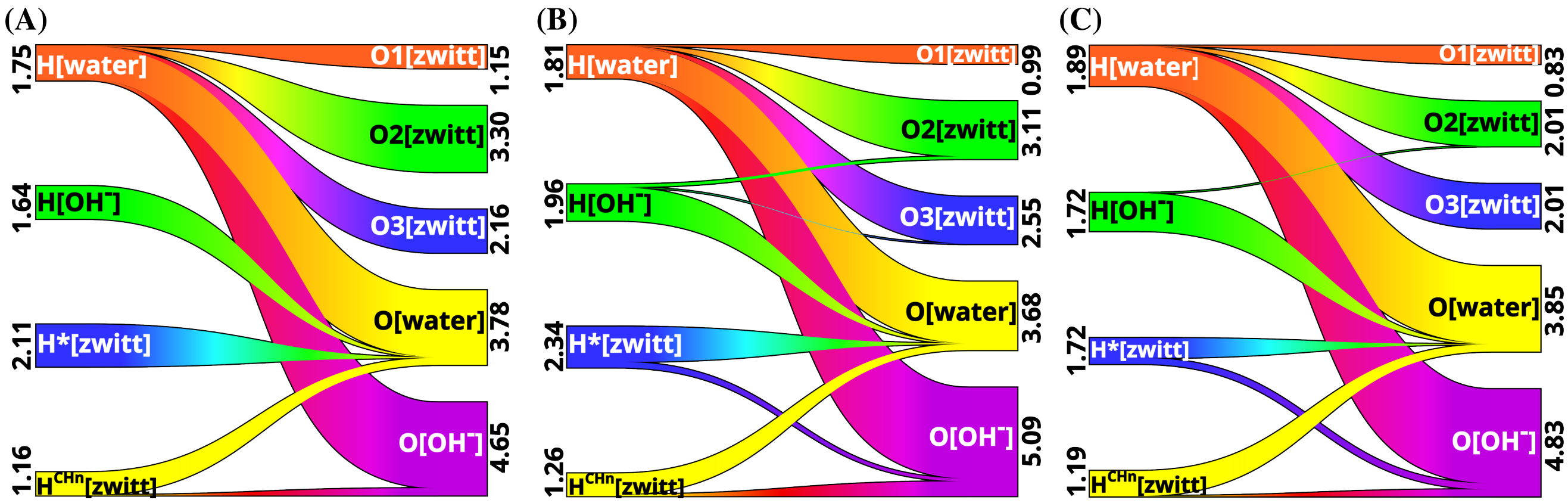

Figure S4: Sankey diagrams showing the hydrogen bond topology during the WTMD simulations starting with conformer **DFT-C1** (**A**), conformer **DFT-C2** (**B**), and conformer **DFT-C3** (**C**). The groups on the left side correspond to the donor sites and the groups on the right side correspond to the acceptor sites. The RGB color scale represents both the intensity and distance of the first maximum in corresponding RDF: Red (very large maximum height and very small distance); Blue (very minimum height and very small distance); Yellow (very large maximum height and very large distance); White (very minimum height and very large distance). The labels are referred as (see the atom numbers in Scheme 2): **H[water]**: Hydrogen atoms from water molecules; **H[OH⁻]**: Hydrogen atom from hydroxyl ions; **H*[zwitt]**: Hydrogen atom to be abstracted from zwitterionic intermediate; **H^{CHn}[zwitt]**: Hydrogen atoms from CHn groups of the zwitterionic intermediate; **O_1,O_2,O_3[zwitt]**: Oxygen atoms 1, 2, and 3 of the zwitterionic intermediate; **O[water]**: Oxygen atom from water molecules; **O[OH⁻]**: Oxygen atom from hydroxyl ions.

S3.3 Time-dependent RDF

Analyzing the time-dependent RDFs between O3 and water hydrogens, g(r)[O3-Hsolv], in Figure S5 (left side) we can see that in the simulation of conformer **DFT-C1**, the O3 interact mostly via HB (second peek in the RDF in Figure S5A). Protonated O3, indicated by the first peek, is found only about 25 ps of simulation. On the other hand, in the simulations of the conformers **DFT-C2** and **DFT-C3**, the O3 is found to be protonated during almost the whole trajectory as showed in Figures S5C and S5E, respectively. This result is interesting because it shows that, in general, the protonation of O3 is highly favored in water and must occur before the deprotonation of the C1 atom.

Additionally, the protonation of the oxygen atom seems to be dependent on the conformation of the zwitterionic intermediate. Thus, the reaction mechanism could differ from the proposal reported in the literature, in which the proton bonded to C1 is transferred either intramolecularly or intermolecularly via the solvent. However, this must obviously depend on the chemical reaction environment and the solvation of the zwitterionic, since the approach of the solvent molecules to the oxygen atom may be more or less facilitated depending on their size, and the hydrogen lability will depend on the basicity of the solvent. Furthermore, an analysis of Figures S5B, S5D, and S5F reveals that the water molecules interact with the hydrogen atom, which is prone to abstraction (labeled as H*), with an average distance of 250 pm. Nonetheless, the deprotonation is not observed.

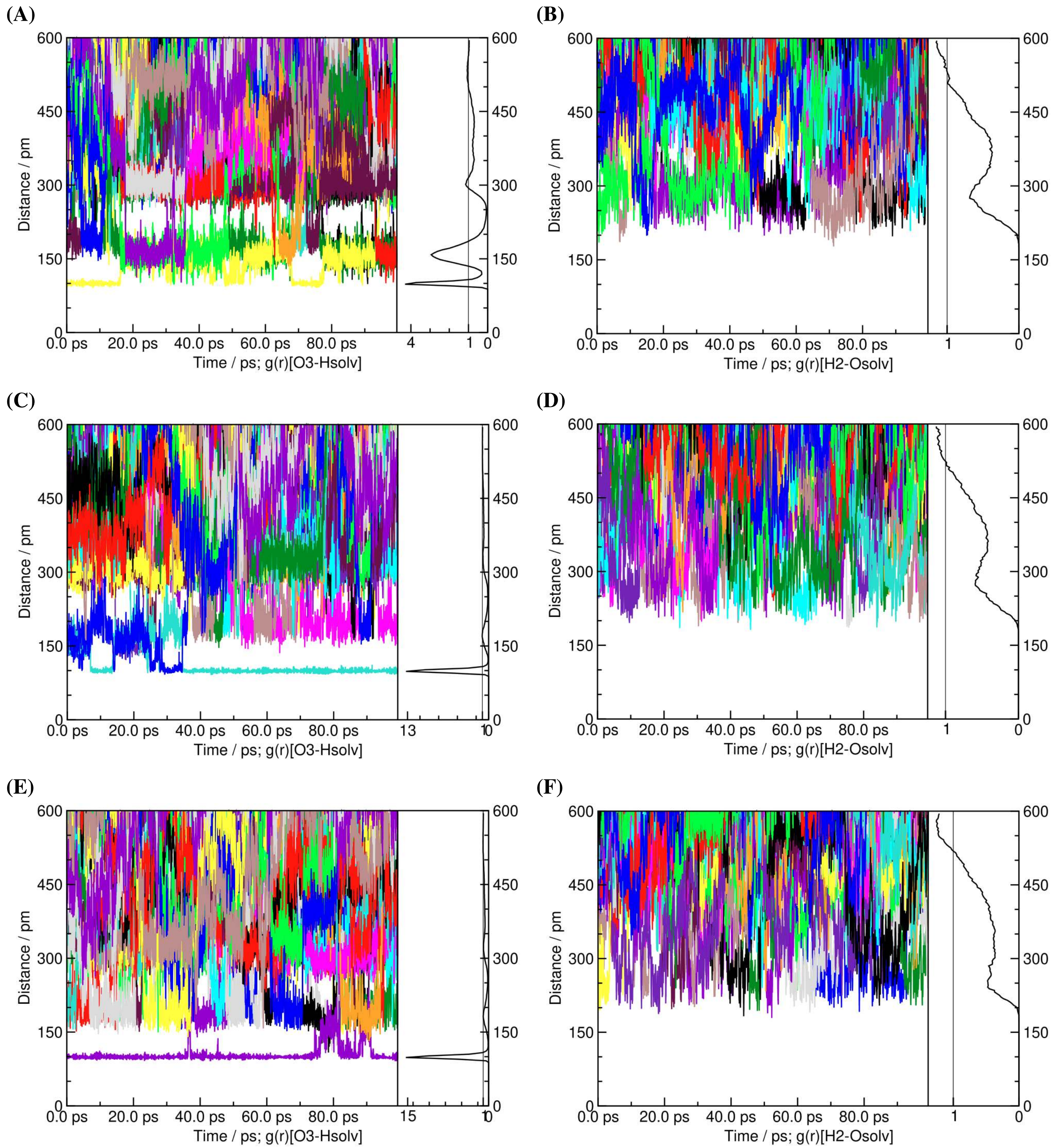

Figure S5: Combined distance time development and radial distribution function between oxygen atom 3 from zwitterionic intermediate and hydrogen atom from solvent (left side), and hydrogen H* from zwitterionic intermediate and oxygen atom from solvent (right side) during the AIMD simulations starting with conformers **DFT-C1** (**A** and **B**), **DFT-C2** (**C** and **D**), and **DFT-C3** (**E** and **F**). The coloured lines represent different interacting O and H atoms of the solvent.

S3.4 Spatial Distribution Function

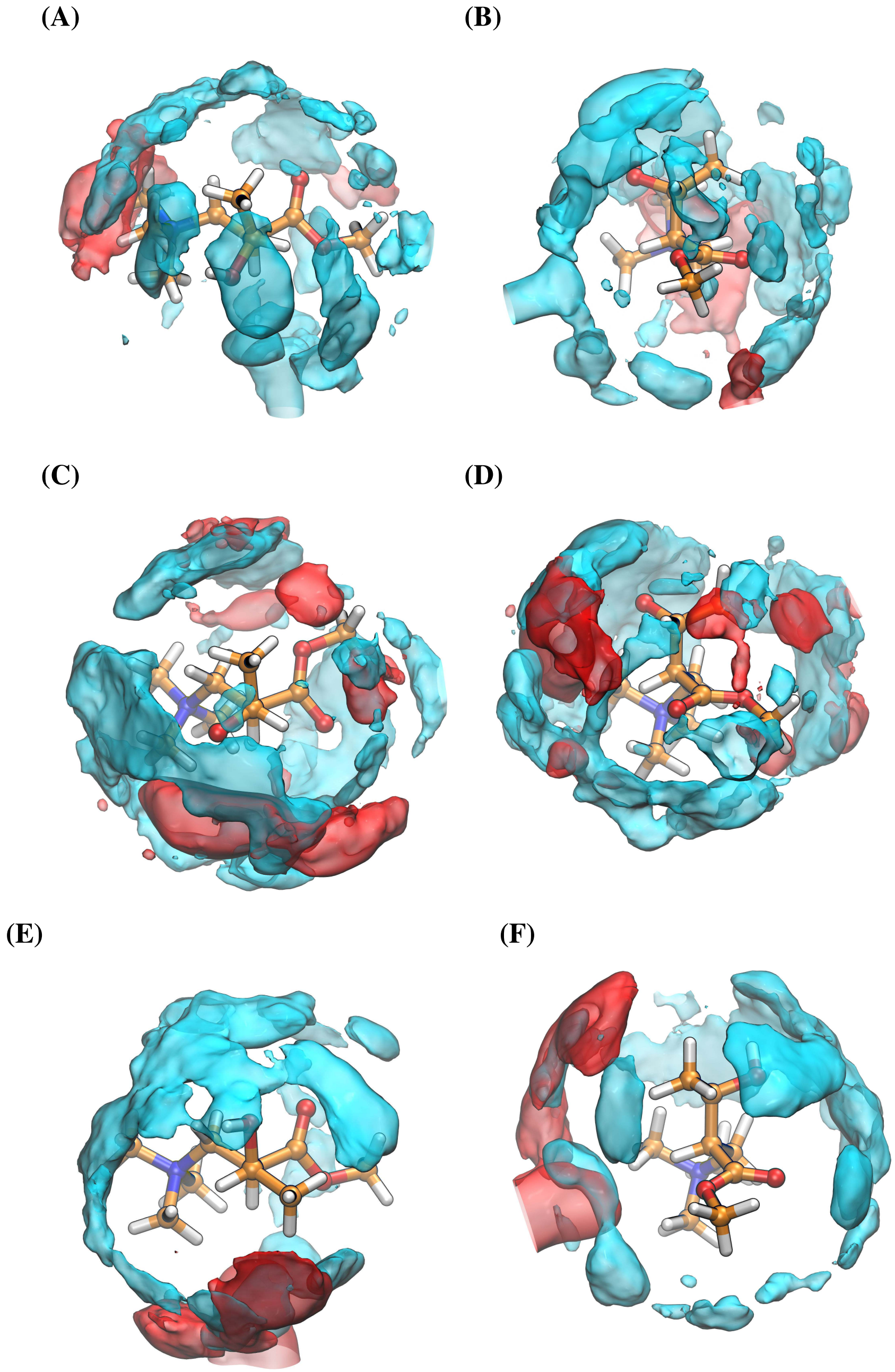

Figure S6: Spatial distribution function (SDF) showing the regions of high probability for finding water molecules (blue color) and the hydroxyl ions (red color) surrounding zwitterionic intermediate structure during the WTMD simulations starting with conformer **DFT-C1** (**A, B**), conformer **DFT-C2** (**C, D**), and conformer **DFT-C3** (**E, F**). Left side corresponds to the top-view and right side to the side-view. The SDF was generated by TRAVIS program and visualized by VMD. The atom colors are: H = white; C = orange; N = blue; O = red.

S4 Assessment of WTMD simulations

In order to ensure the reliability of the WTMD simulations and to ascertain the accuracy of the free energy profile, Figure S7 depicts the Gaussian height and CV evolution over time. The evolution of Gaussian height is regular as it can be seen in Figures S7A, S7C, and S7E. Discontinuities in the decay of the Gaussian height are frequent and coincide with the proton exchanges between the zwitterionic and solvent molecules, which are repeatedly sampled. The CV evolution shown in Figures S7B, S7D, and S7F reveals that the C-H bond is definitively broken, towards resulting in the deprotonated zwitterionic, around 25-26 ps for the simulations of conformers **DFT-C1** and **DFT-C2**, while for conformer **DFT-C3** this event occurs around 60 ps. The CV region between 0.2 and 0.4 corresponds to interactions between the deprotonated carbon of the zwitterionic intermediate and nearby solvent hydrogens. It is short-lived hydrogen bond interactions that lead to fluctuations in the coordination number instead of keeping it near zero when the carbon is bare. This behavior reflects a dynamically sampled region rather than a stable intermediate, and the reweighting procedure averages over these fluctuations, resulting in a smooth free energy profile without a distinct minimum (see Figure S8).

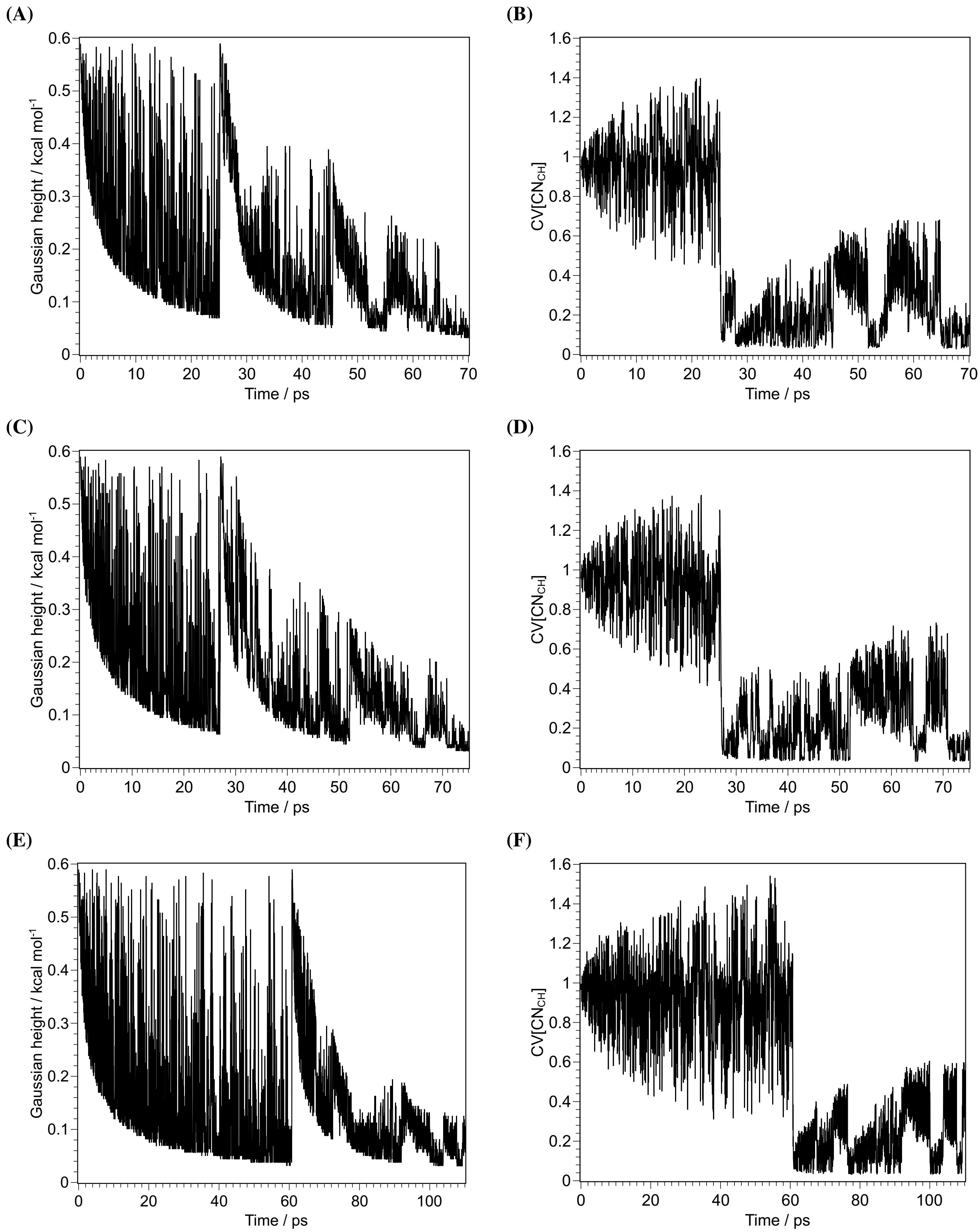

Figure S7: Evolution of the Gaussian height (left side) and CV with time (right side) for simulations starting with conformers **DFT-C1** (**A** and **B**), **DFT-C2** (**C** and **D**), and **DFT-C3** (**E** and **F**).

S5 Thermodynamic Analysis of WTMD simulations

The free-energy profile along the C-H coordination number was analyzed using the energy-entropy decomposition formalism introduced by Gimondi et al.[13]. The Helmholtz free-energy $\Delta F(s)$ was reconstructed by reweighting the well-tempered metadynamics trajectory in the NVT ensemble, explicitly accounting for the time-dependent bias potential. The internal-energy contribution $\Delta U(s)$ was obtained as the conditional ensemble average of the instantaneous potential energy at fixed values of the collective variable, while the entropic contribution was evaluated as $-T\Delta S(s) = \Delta F(s) - \Delta U(s)$.

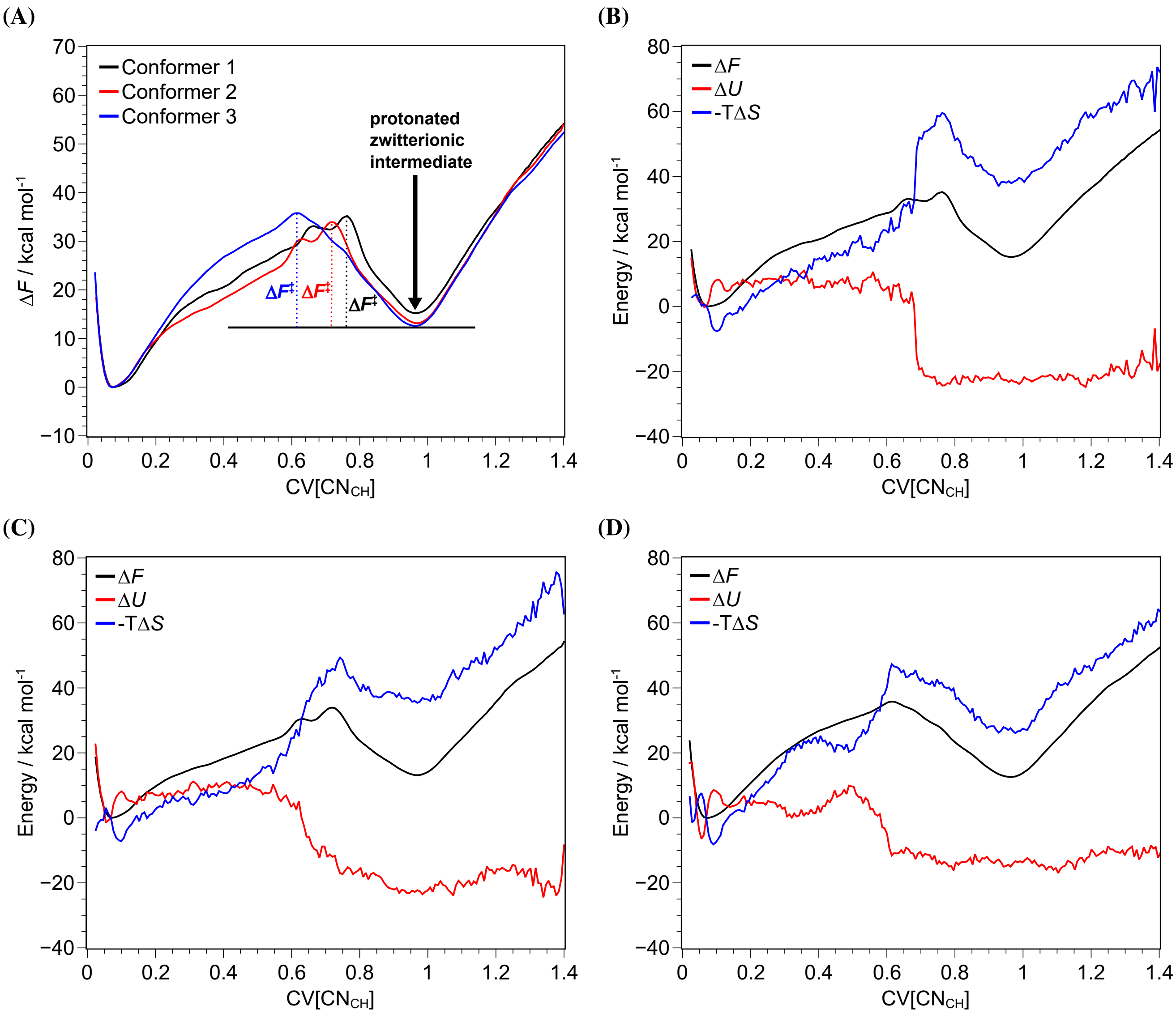

Figure S8: **(A)** One-dimensional free-energy (F) profile as a function of the single collective variable (coordination number; CV[CN_{CH}]), obtained from well-tempered metadynamics simulations. The $\Delta F^{\ddagger}$ corresponds to the proton dissociation barrier. Free energy decomposition of the MBH proton transfer pathway in terms of internal energy, U, and entropy, S, as a function of the CV[CN_{CH}], for simulations starting with conformer **DFT-C1** (**B**), conformer **DFT-C2** (**C**), and conformer **DFT-C3** (**D**). The energies are relative to the deprotonated zwitterionic intermediate.

S6 Natural Localized Molecular Orbitals analysis

Table S2: Characterization of main delocalized bonding orbitals from the RTS of the WTMD simulations starting with conformers **DFT-C1**, **DFT-C2**, and **DFT-C3** in terms of the contribution from different atomic hybrid orbitals and *s*/*p*/*d*/*f* character.[a] See the solute molecule labels in Figure S2 and representations of orbital isosurfaces in Figure 5.

Isosurface	NLMO	Atomic Hybrid Contributions
	σC1-H*	68 C1 $s(17)p(83)$
		26 H* $s(100)$
		2 C2 $s(0.4)p(98.6)d(1)$
		1 O2 $s(5.5)p(94.0)d(0.5)$
		1 C4 $s(0.5)p(97.1)d(2.4)$
		0.3 C3 $s(0.5)p(97.1)d(2.3)f(0.1)$
A-I		0.2 N $s(57.2)p(41.4)d(1.3)f(0.1)$
	LP_3O^{top}	90 O $s(39)p(61)$
		8 H[O3] $s(99.7)p(0.3)$
		2 O3 $s(34.1)p(65.8)d(0.1)$
	LP_2O^{bottom}	94 O $s(20)p(80)$
		4 H* $s(99)p(1)$
		1 C1 $s(22.8)p(77.0)d(0.1)$
	LP_2O	95 O^{top} $s(15)p(85)$
A-II		4 H $s(99.7)p(0.3)$
		1 O^{bottom} $s(34)p(65.8)d(0.3)$
	LP_1O	97 O $s(24)p(76)$
A-III		2 H_{water} $s(99.5)p(0.5)$
		1 O_{water} $s(36.4)p(63.2)d(0.4)$
	LP_1O3	98 O3 $s(35)p(65)$
		1 C3 $s(5)p(92.7)d(2.2)f(0.2)$
		0.4 C1 $s(33.4)p(64.6)d(1.7)f(0.3)$
		0.4 H_{water} $s(99.2)p(0.7)d(0.1)$
		0.3 H_{water} $s(98.7)p(1.2)d(0.1)$
A-IV	LP_2O3	95 O3 $s(13)p(87)$
		2 H_{water} $s(99.5)p(0.5)$
		1 C3 $s(6)p(89.8)d(4)f(0.2)$
		1 O_{water} $s(33.7)p(66.2)d(0.1)$
		0.4 H[C3] $s(98.9)p(0.8)d(0.3)$
		0.2 C1 $s(48.4)p(47.7)d(3.3)f(0.7)$
	σC1-H*	65 C1 $s(17.3)p(82.6)d(0.1)$
		31 H* $s(100)$
		2 C2 $s(0.6)p(98.5)d(0.9)$
		1 O2 $s(3.7)p(96)d(0.2)$
		0.4 C3 $s(2.1)p(95.7)d(2)f(0.2)$
B-I		0.3 C4 $s(0.1)p(96.8)d(3)f(0.1)$
		0.3 O1 $s(0.3)p(98.5)d(1.2)$
		0.1 N $s(57.1)p(41.4)d(1.3)f(0.2)$

Table S2 continued from previous page

Isosurface	NLMO	Atomic Hybrid Contributions
	LP_2O	98 O *s*(23.9)*p*(76)*d*(0.1)
		1 H* *s*(99.1)*p*(0.8)*d*(0.1)
		0.5 C1 *s*(23.3)*p*(76.5)*d*(0.1)*f*(0.1)
B-II	LP_2O	96 O *s*(11)*p*(89)
		2 H_{water} *s*(99.4)*p*(0.5)*d*(0.1)
		1 C3 *s*(1.6)*p*(94.6)*d*(3.6)*f*(0.2)
		0.5 O_{water} *s*(29.7)*p*(70)*d*(0.2)
		0.5 C1 *s*(47.4)*p*(50.6)*d*(1.6)*f*(0.4)
		0.3 C5 *s*(35.2)*p*(60.8)*d*(3.1)*f*(1)
B-III	$LP_1O_{OH^-}$	98 O *s*(52)*p*(48)
		1 H[O3] *s*(99)*p*(1)
		0.4 O3 *s*(46)*p*(53.4)*d*(0.5)
B-IV	$LP_2O_{OH^-}$	97 O *s*(1.4)*p*(98.5)*d*(0.1)
		1 H_{water} *s*(99)*p*(1)
		1 H_{water} *s*(99)*p*(1)
		0.4 O_{water} *s*(33.6)*p*(66.3)*d*(0.1)
		0.2 O_{water} *s*(27)*p*(72.7)*d*(0.3)
B-V	$LP_3O_{OH^-}$	92 O *s*(24)*p*(76)
		6 H_{water} *s*(99.8)*p*(0.2)
		1.5 O_{water} *s*(29.3)*p*(70.5)*d*(0.2)
C-I	σC1-H*	74 C1 *s*(19.4)*p*(80.6)
		21 H* *s*(100)
		1.4 C2 *s*(2.9)*p*(95.7)*d*(1.4)*f*(0.1)
		0.8 C4 *s*(2.6)*p*(95.3)*d*(2.1)
		0.5 O2 *s*(20.3)*p*(79)*d*(0.7)
		0.4 C3 *s*(1.8)*p*(94.5)*d*(3.6)*f*(0.1)
		0.4 H[C3] *s*(98.5)*p*(1.3)*d*(0.2)
		0.3 N *s*(61)*p*(38.2)*d*(0.6)*f*(0.1)
	LP_2O	89 O *s*(32.1)*p*(67.8)*d*(0.1)
		8 H* *s*(99.5)*p*(0.5)
		2 C1 *s*(24.8)*p*(75.1)*d*(0.1)
C-II	LP_2O3	96 O3 *s*(28)*p*(72)
		2 H_{water} *s*(99.4)*p*(0.5)*d*(0.1)
		0.5 O_{water} *s*(22.9)*p*(76.7)*d*(0.4)
		0.5 C3 *s*(6)*p*(89.4)*d*(4.4)*f*(0.2)
		0.3 C1 *s*(52.7)*p*(44.6)*d*(2.1)*f*(0.6)
C-III	$LP_1O_{OH^-}$	98 O *s*(36)*p*(64)
		1 H_{water} *s*(99.6)*p*(0.4)
		0.4 H_{water} *s*(99.2)*p*(0.7)*d*(0.1)
		0.3 O_{water} *s*(29.1)*p*(70.8)*d*(0.1)
C-IV	$LP_2O_{OH^-}$	96 O *s*(32)*p*(68)
		2.5 H_{water} *s*(99.6)*p*(0.3)
		0.6 H_{water} *s*(99.3)*p*(0.7)
		0.6 O_{water} *s*(28)*p*(72)

Table S2 continued from previous page

Isosurface	NLMO	Atomic Hybrid Contributions
C-V	$LP_3O_{OH^-}$	94 O *s*(9.5)*p*(90.5)
		3 H_{water} *s*(99.6)*p*(0.3)
		0.8 H_{water} *s*(99.5)*p*(0.4)
		0.8 O_{water} *s*(26.3)*p*(73.6)
		0.7 H_{water} *s*(99.3)*p*(0.6)
		0.2 O_{water} *s*(30.4)*p*(68.5)*d*(1.1)
		0.2 O_{water} *s*(35.6)*p*(63.4)*d*(1)

[a] All values are in %.

S7 On-Flow NMR experiments

Kinetic experiments were performed using an NMR-based monitoring approach. The goal was to implement a sampling strategy capable of providing detailed kinetic profiles and capturing both intermediates and products. Initial attempts *in situ* monitoring (reaction conducted directly in the NMR tube) were ineffective due to the need for continuous stirring. Therefore, an *on-flow* sampling method was adopted, allowing efficient monitoring while preserving reaction homogeneity. The experimental data presented in Figure 7B were obtained using an *on-flow* NMR setup. A Shimadzu LC-10ADVP peristaltic pump was used to circulate the reaction mixture from the reaction vessel to the 5 mm NMR tube *via* a PEEK tubing line. The temperature of the flow system was maintained at 25 °C by means of a thermostatic bath connected to a jacketed reaction vessel and a rubber tube that externally covered the entire length of the transfer line. Sterring was maintained constant at 550-600 rpm by means of a magnetic stir plate during all experiments.

Reactions were performed using a total solvent volume of 40 mL, consisting of 20 mL of water and 20 mL of acetonitrile. It is noteworthy that acetonitrile was added to improve the solubility of benzaldehyde. Furthermore, since the reaction occurs through a different mechanism in a non-protic solvent with a higher energy barrier, the reaction takes place via the protic solvent route, and acetonitrile has no influence. Tetramethylsilane (TMS) was added as an internal reference for the NMR spectra. The reactant concentrations were 0.25 mol L^{-1} for both DABCO (catalyst) and methyl acrylate, and 0.125 mol L^{-1} for the corresponding aldehyde. The flow system was connected to a Bruker Avance III 9.4 T NMR spectrometer (400 MHz for ^{1}H) equipped with a 5 mm BBI inverse detection probe. Each reaction was monitored over approximately 58 hours, acquiring one spectrum every 5 minutes. For each spectrum, 16 scans were recorded with a relaxation delay of 15 seconds, resulting in a total of 735 kinetic data points. The peristaltic pump was set to a constant flow rate of 1 mL min^{-1} throughout the monitoring period.

The pulse sequence used for kinetic monitoring was adapted from a sequence originally developed for LC-NMR applications,[14] including WET solvent suppression block[15] to saturated solvent signals, as the large solvent volumes required for flow monitoring precluded the use of deuterated solvents. In these experiments, water (~4.3 ppm) and acetonitrile (~2.1 ppm) signals were selectively suppressed. Due to the absence of a lock field resulting in the spectral drift, leading to shifts in signal frequencies, the pulse sequence was modified to include an automatic pre-acquisition scan at each kinetic time point. This scan identified the most intense signals and dynamically adjusted the solvent suppression parameters based on previously optimized pulse power.

After assembling the flow system, the solvents were added to the reaction flask along with DABCO. At this stage, probe tuning, receiver gain, and magnetic field homogeneity were adjusted. Following optimization of the solvent suppression pulses power, methyl acrylate was added, followed by the aldehyde. This order of addition was chosen to avoid pH changes that could result from the late addition of the DABCO (basic specie). After monitoring each reaction, the acquired spectra were processed and integrated across all 735 kinetic time points. The spectral regions selected for integration corresponded to the DABCO signal at 2.7 ppm, methyl acrylate between 6.3-6.5 ppm, the aldehyde between 9.9-10.0 ppm, the intermediate (**1**) between 6.1-6.2 ppm, and the product signal when observed. Based on these integrations, kinetic profiles were constructed, as exemplified in Figure S9 for the MBH reaction with pivaldehyde (**R1**). The relative intensities shown in the plots correspond to the signal areas of each analyte, normalized to the DABCO signal.

In Figure S9, a decrease in the signal intensities of both methyl acrylate and the aldehyde is observed over time, following by the formation of intermediate (**1**). This suggests that, once intermediate (**1**) is formed (as observed by ^{1}H NMR spectra) by the addition of DABCO to the olefin, a subsequent

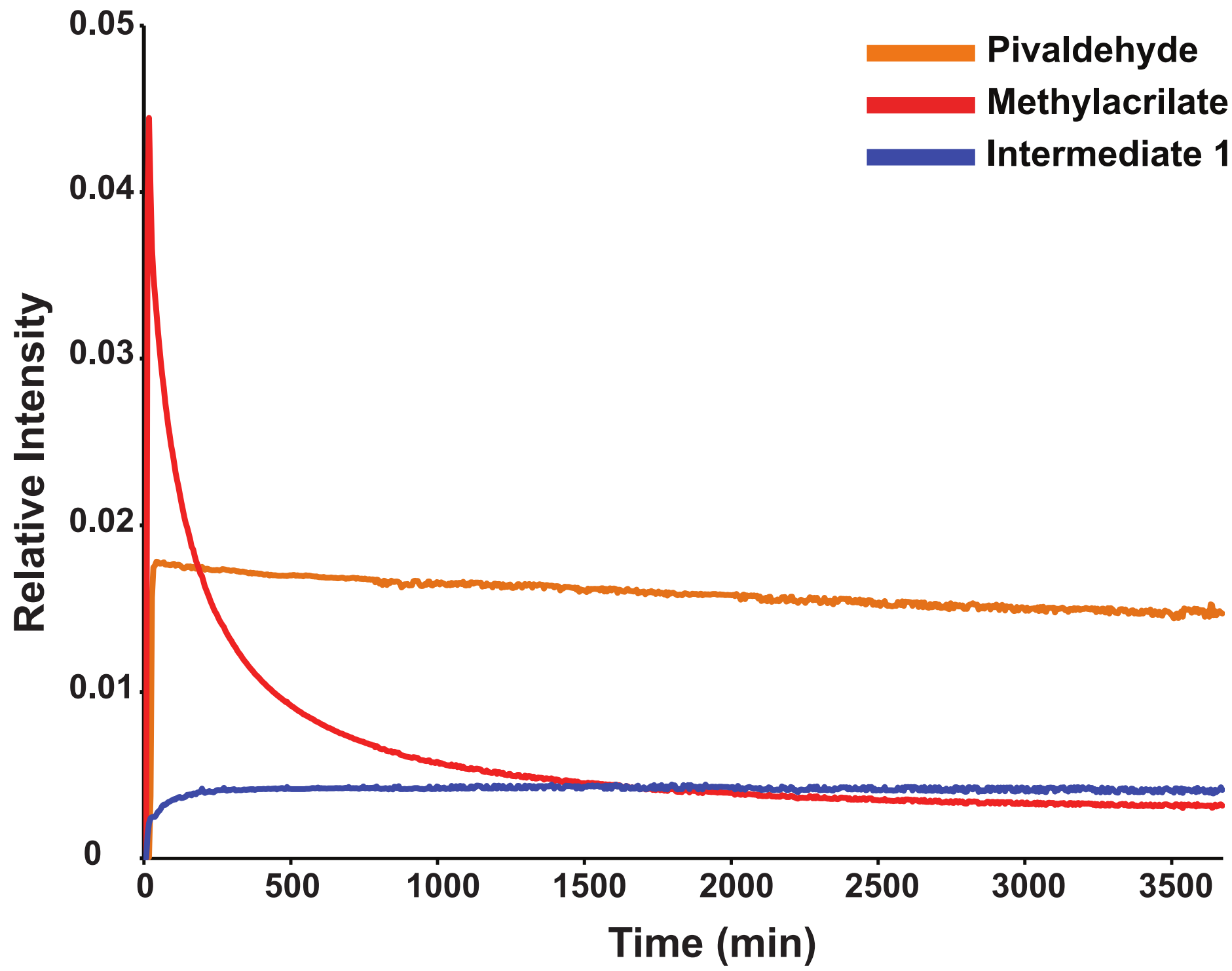

Figure S9: Kinetic monitoring profile for the MBH reaction with pivaldehyde (R1) as the substrate, showing the time-dependent variation in the relative intensities of the aldehyde, methyl acrylate, and intermediate (**1**), normalized to the DABCO signal.

rearrangement could occur, leading to the transient species (**2**), which exists in solution prior to the hydrogen abstraction step that forms the final MBH adduct. Although intermediate (**2**) is not directly monitored in the NMR spectra, because its signals overlap with those of intermediate **1**, its presence is inferred from the reaction kinetics, particularly the consumption of pivaldehyde. This observation is particularly relevant, as the study focuses on the influence of the solvent in the hydrogen abstraction step. The theoretical data are consistent with the experimental results, which indicate that the steric hindrance introduced by the isopropyl group significantly slows down the hydrogen abstraction and subsequent MBH adduct was not detected during the monitoring period (Figure S10). In the case of

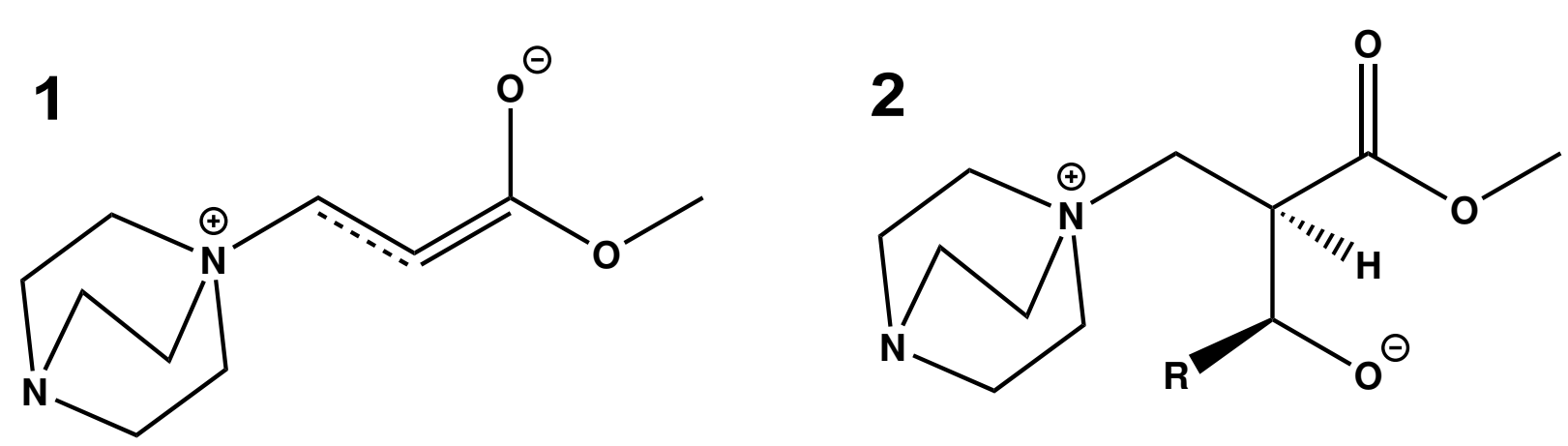

R1: Isopropyl
R2: Methyl
R3: Aryl

Scheme S1: MBH reaction intermediates formed by means of a conjugated addition of a tertiary amine to methyl acrylate (**1**) and the reaction between **1** and an aldehyde (**2**).

less hindered aldehydes bearing methyl or aryl substituents, the formation of the MBH adducts was observed. This is evidenced by the kinetic monitoring plots for the MBH reactions of acetaldehyde (**R2**)

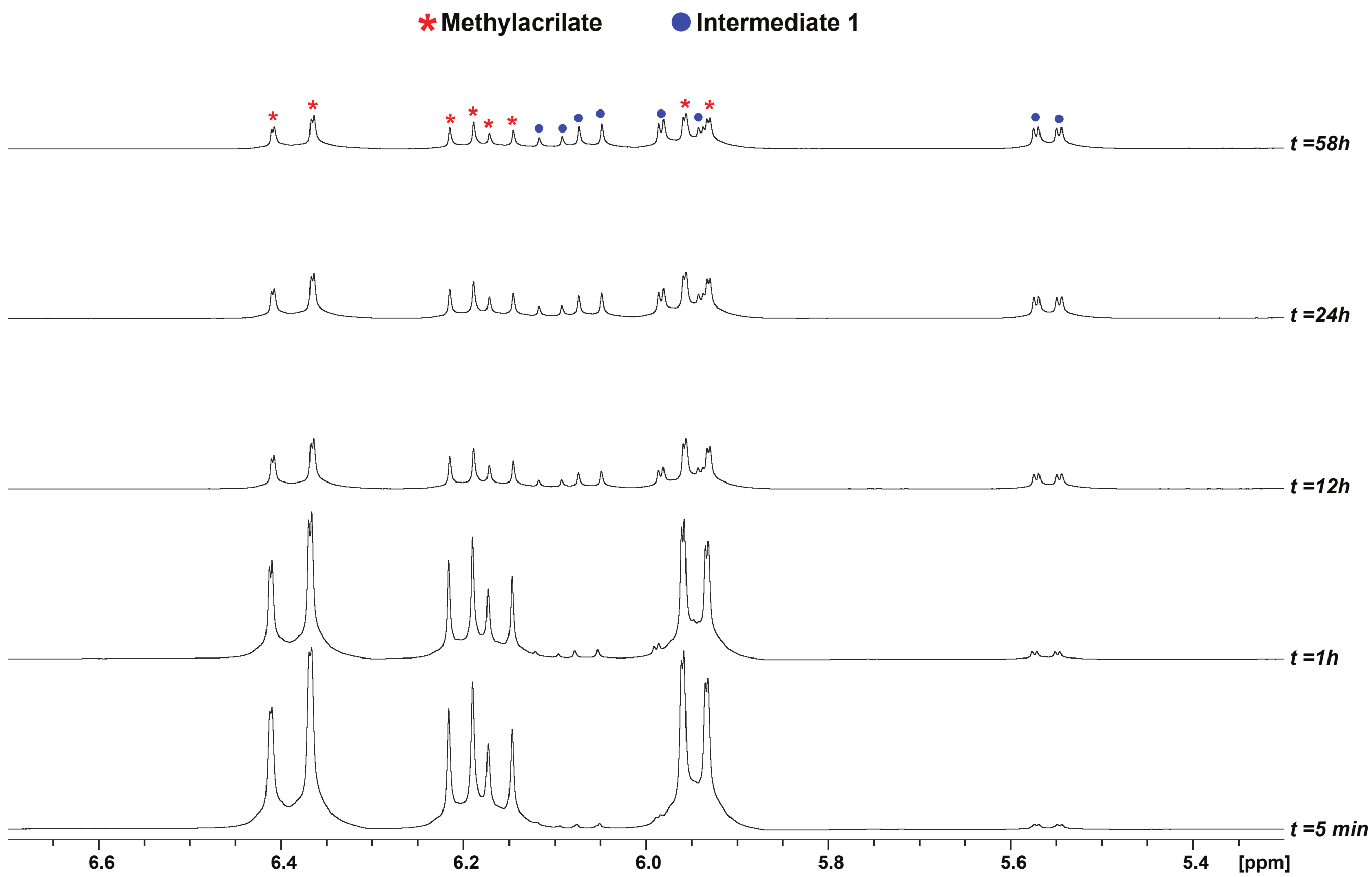

Figure S10: Stacked ^{1}H NMR spectra from the kinetic monitoring of the MBH reaction with pivaldehyde (**R1**), highlighting the olefinic hydrogen region. The marked signals correspond to the intermediate (green) and methyl acrylate (red) at the indicated reaction times.

and benzaldehyde (**R3**), shown in Figures S11A and S11B,respectively.

It can be observed a decreasing in the relative intensity of methyl acrylate and increasing of both the intermediate and the MBH adduct for both aldehydes (Figure S11). These trends are further supported by the NMR spectra shown in Figure S12 for the acetaldehyde adduct and Figure S13 for the benzaldehyde adduct.

^{1}H-^{13}C HSQC experiments were performed on aliquots collected at the end of the kinetic monitoring period to confirm the assignment of the observed signals to the MBH adducts. Figure S14 shows the HSQC contour plot obtained from the reaction with acetaldehyde, highlighting the region where the geminal hydrogens correlate with a carbon signal at 117.25 ppm. The assignment of the adduct structure was also supported by the observed triplet multiplicity of the geminal hydrogens, which results from both the ^2J coupling between them and the ^4J coupling with the carbinolic hydrogen. Since the ^2J and ^4J coupling constants are of similar magnitude, they give rise to the triplet pattern. The same evidence was observed in the HSQC spectrum when benzaldehyde was used as the substrate (Figure S15)

The potential energy profiles shown in Figure 7A are consistent with the experimental kinetic results, indicate that the formation of the adduct from the methyl-substituted aldehyde occurs slightly faster than that from the aryl-substituted aldehyde.

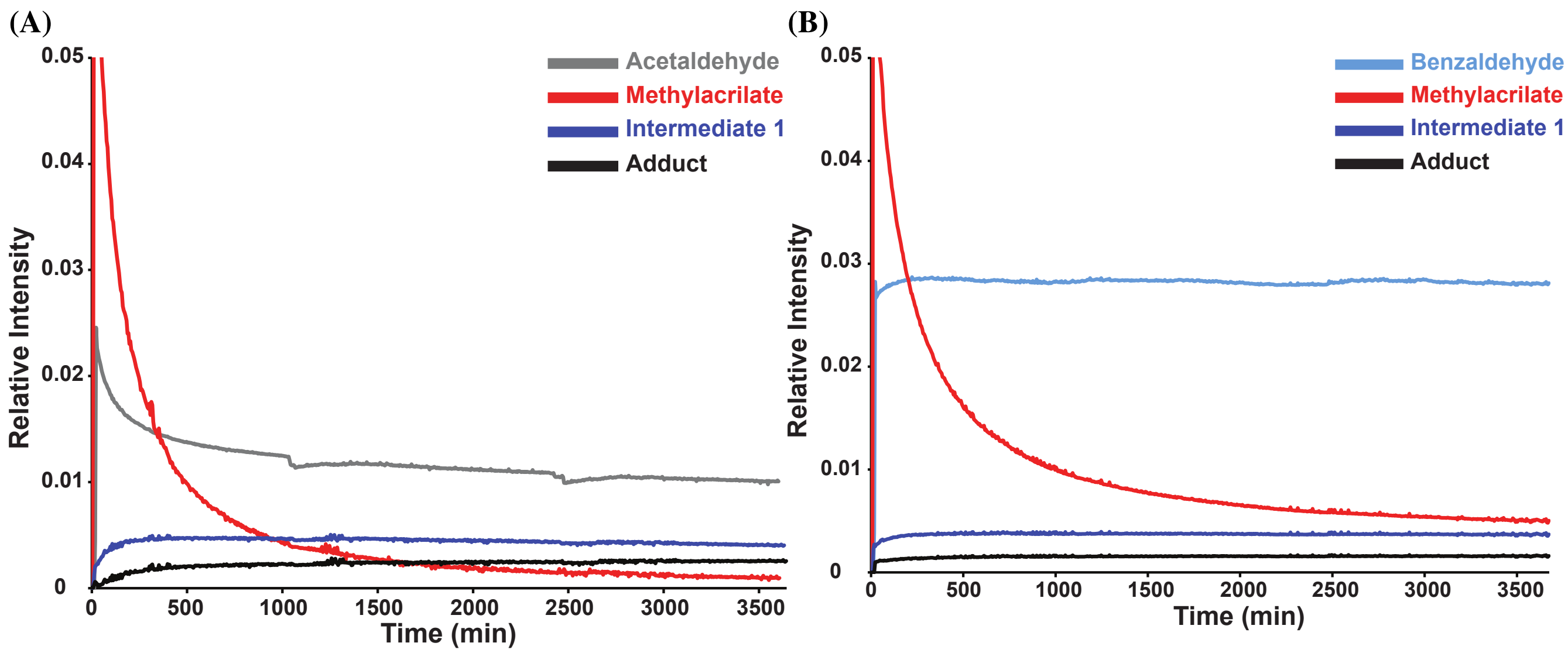

Figure S11: Kinetic monitoring profiles for the MBH reactions using (**A**) acetaldehyde (R2) and (**B**) benzaldehyde (R3) as substrates. The plots show the time-dependent variation in the relative intensities of the aldehyde, methyl acrylate, intermediate, and adduct, normalized to the DABCO signal.

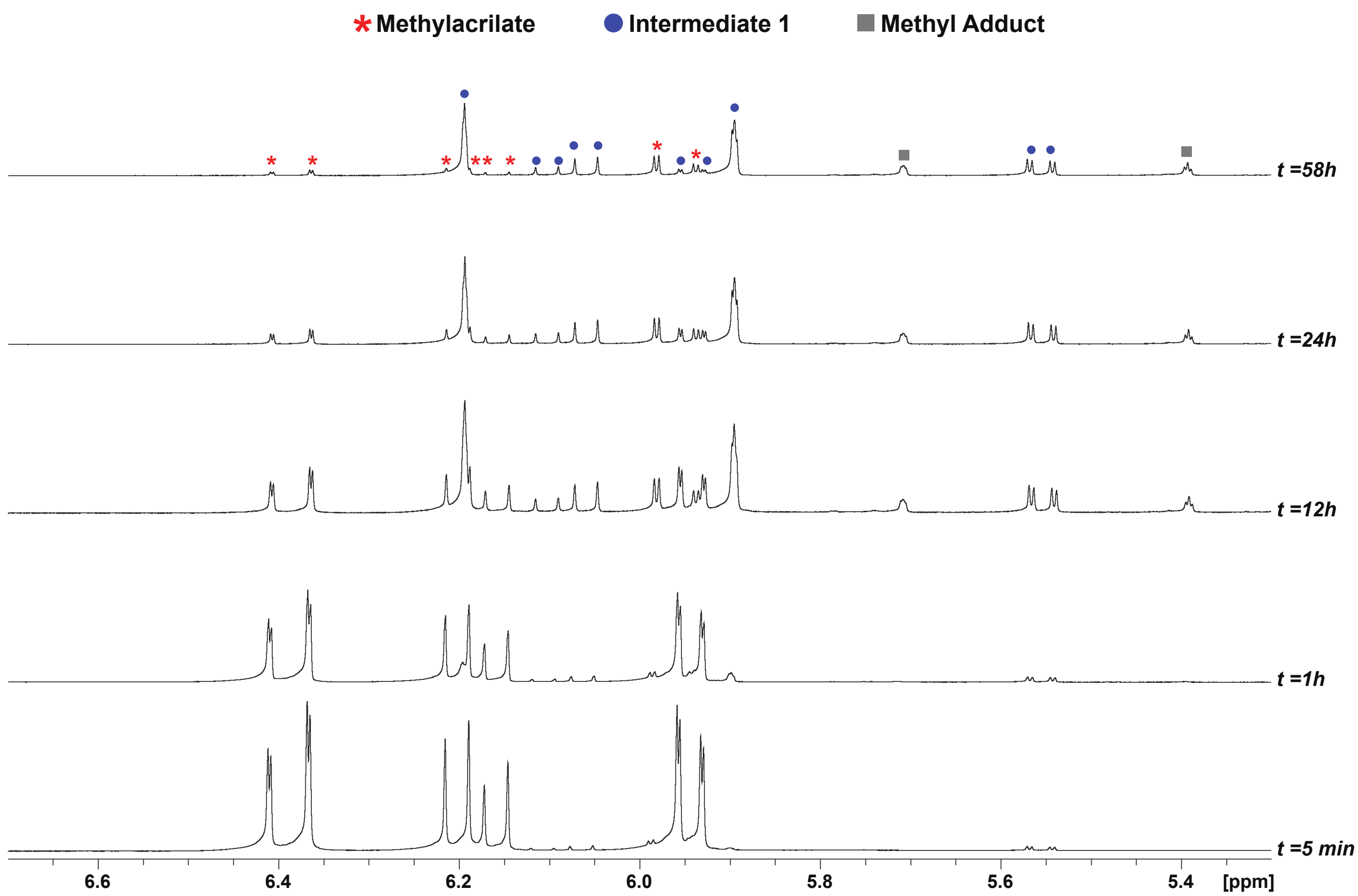

Figure S12: Stacked ^{1}H NMR spectra from the kinetic monitoring of the MBH reaction with acetaldehyde (**R2**), highlighting the olefinic hydrogen region. The marked signals correspond to the intermediate (green), methyl acrylate (red), and the MBH adduct (gray) at the indicated reaction times.

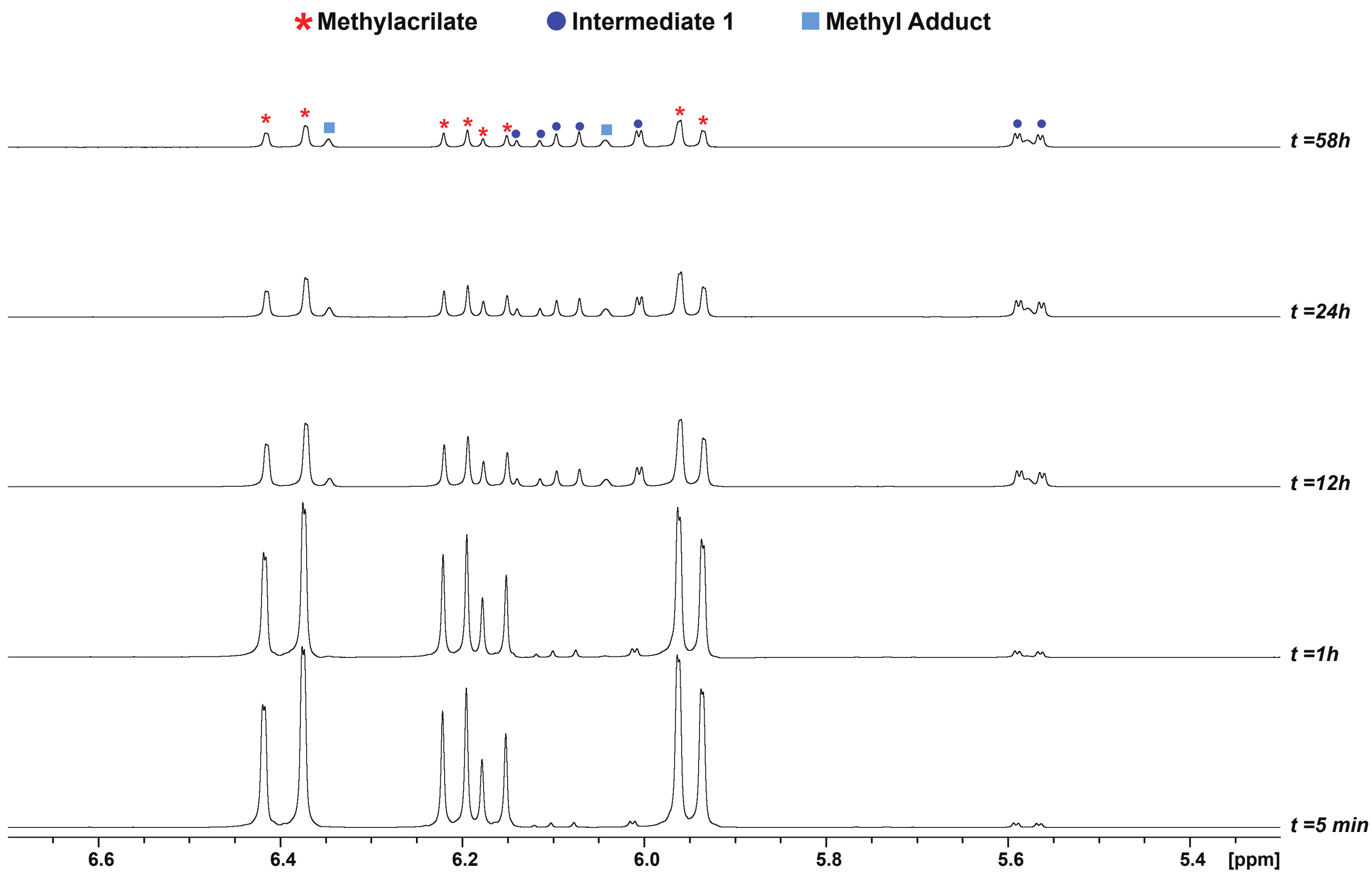

Figure S13: Stacked ^{1}H NMR spectra from the kinetic monitoring of the MBH reaction with benzaldehyde (**R3**), highlighting the olefinic hydrogen region. The marked signals correspond to the intermediate (green), methyl acrylate (red), and the MBH adduct (blue) at the indicated reaction times.

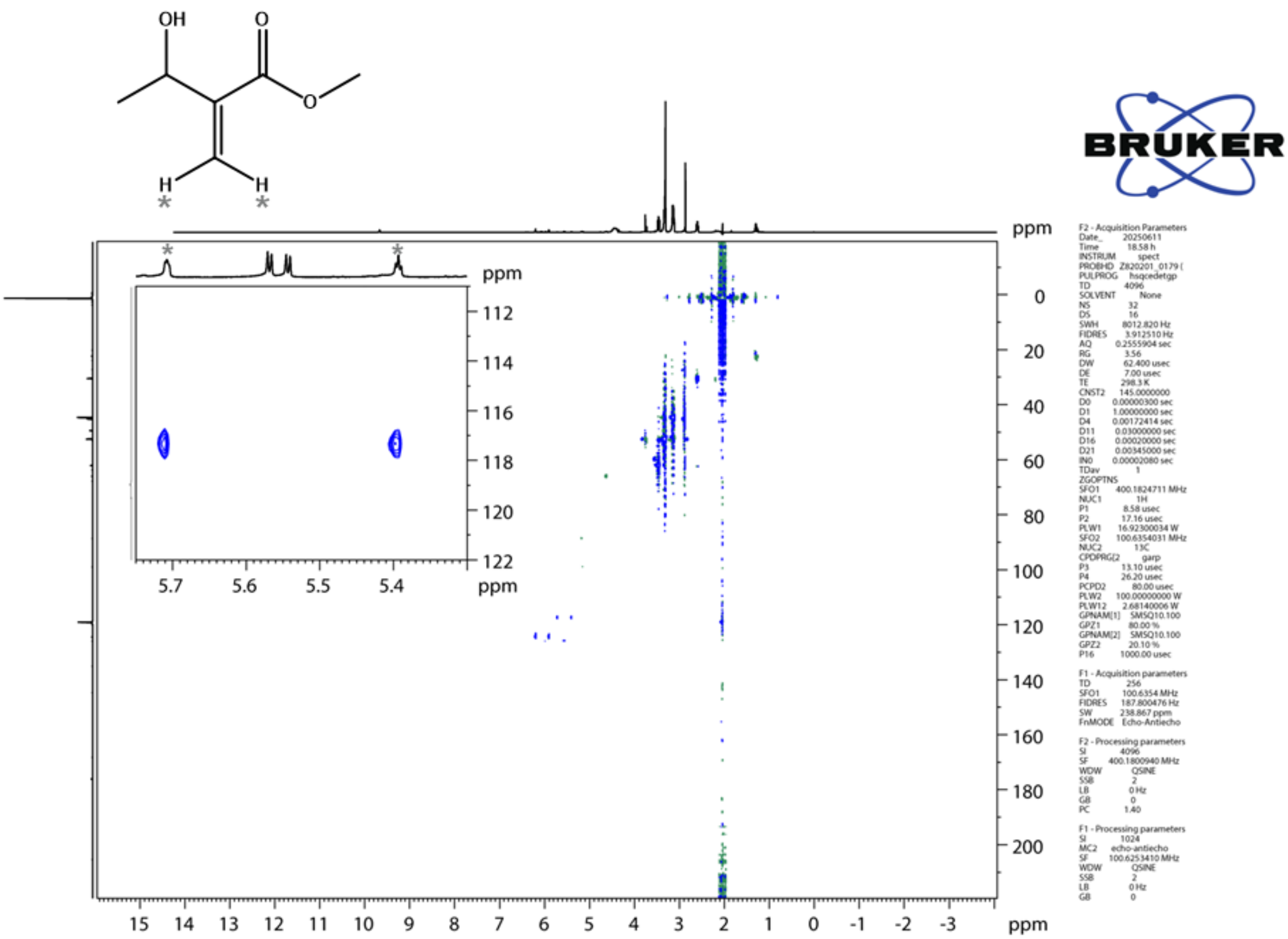

Figure S14: ^{1}H-^{13}C HSQC contour plot of the MBH reaction with acetaldehyde (**R2**). The insert highlights the cross-peaks corresponding to the hydrogens labelled in the adduct structure.

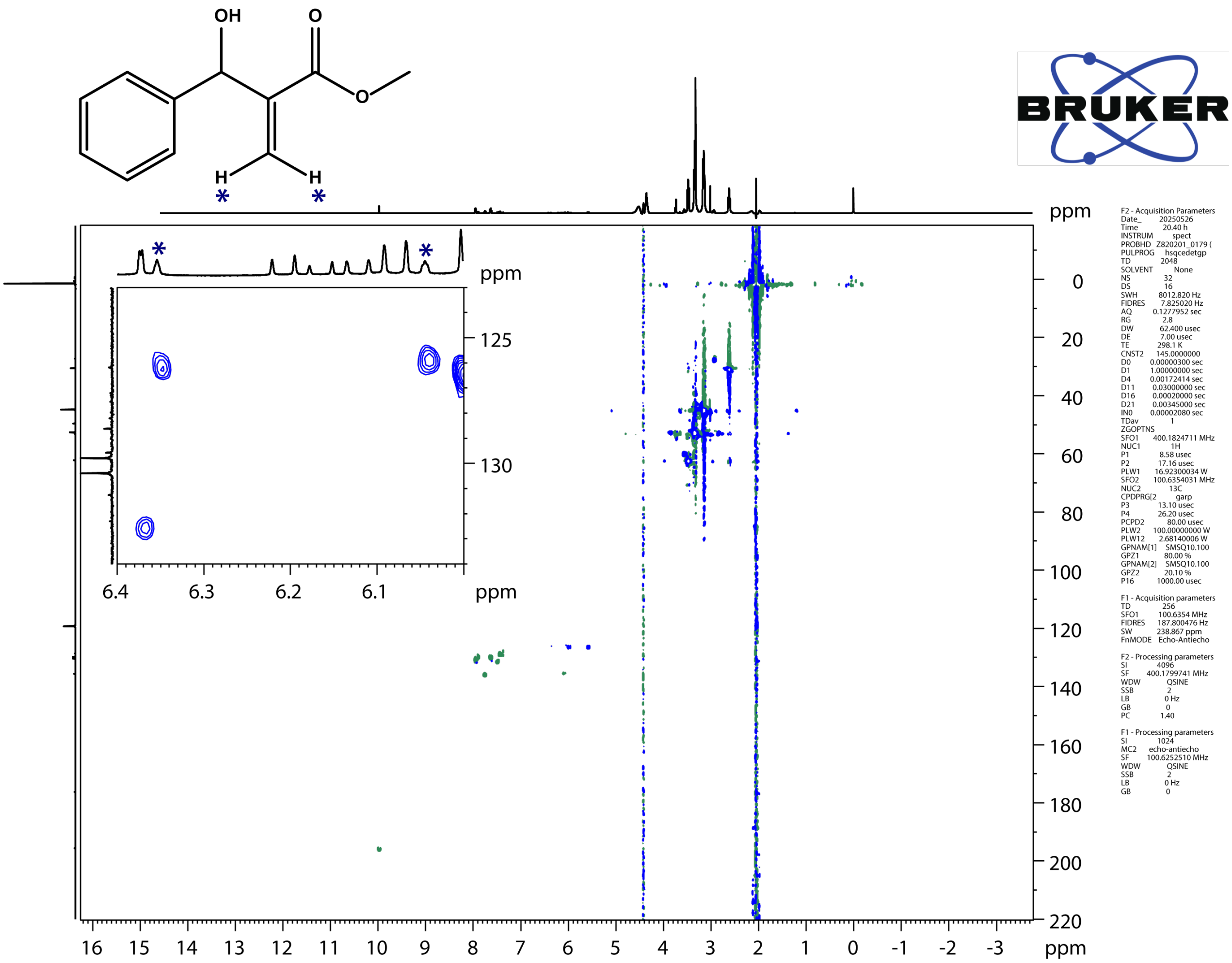

Figure S15: ^{1}H-^{13}C HSQC contour plot of the MBH reaction with benzaldehyde (**R3**). The insert highlights the cross-peaks corresponding to the hydrogens labelled in the adduct structure.

References

(1) DiStasio Jr., R. A.; Santra, B.; Li, Z.; Wu, X.; Car, R. The individual and collective effects of exact exchange and dispersion interactions on the ab initio structure of liquid water. *J. Chem. Phys.* **2014**, *141*, 84502.

(2) Soper, A.; Phillips, M. A new determination of the structure of water at 25 C. *Chem. Phys.* **1986**, *107*, 47–60.

(3) Soper, A. The radial distribution functions of water and ice from 220 to 673 K and at pressures up to 400 MPa. *Chem. Phys.* **2000**, *258*, 121–137.

(4) Hura, G.; Russo, D.; Glaeser, R. M.; Head-Gordon, T.; Krack, M.; Parrinello, M. Water structure as a function of temperature from X-ray scattering experiments and ab initio molecular dynamics. *Phys. Chem. Chem. Phys.* **2003**, *5*, 1981–1991.

(5) Grimme, S.; Ehrlich, S.; Goerigk, L. Effect of the damping function in dispersion corrected density functional theory. *J. Comp. Chem.* **2011**, *32*, 1456–1465.

(6) Morrone, J. A.; Car, R. Nuclear Quantum Effects in Water. *Phys. Rev. Lett.* **2008**, *101*, 017801.

(7) Sorenson, J. M.; Hura, G.; Glaeser, R. M.; Head-Gordon, T. What can x-ray scattering tell us about the radial distribution functions of water?. *J. Chem. Phys.* **2000**, *113*, 9149–9161.

(8) Soper, A. K.; Benmore, C. J. Quantum Differences between Heavy and Light Water. *Phys. Rev. Lett.* **2008**, *101*, 065502.

(9) Kuhne, T. D.; Krack, M.; Parrinello, M. Static and dynamical properties of liquid water from first principles by a novel Car- Parrinello-like approach. *J. Chem. Theory Comput.* **2009**, *5*, 235–241.

(10) Mitchell, M.; Muftakhidinov, B.; Winchen, T.; Jędrzejewski-Szmek, Z.; Badger, T. G.; badshah400, “markummitchell/engauge-digitizer: Version 10.4”, 2017.

(11) Brehm, M.; Kirchner, B. TRAVIS - A Free Analyzer and Visualizer for Monte Carlo and Molecular Dynamics Trajectories. *J. Chem. Inf. Model.* **2011**, *51*, 2007–2023.

(12) Brehm, M.; Thomas, M.; Gehrke, S.; Kirchner, B. TRAVIS—A free analyzer for trajectories from molecular simulation. *J. Chem. Phys.* **2020**, *152*, 164105.

(13) Gimondi, I.; Tribello, G. A.; Salvalaglio, M. Building maps in collective variable space. *J. Chem. Phys.* **2018**, *149*, 104104.

(14) Sidelmann, U. G.; Gavaghan, C.; Carless, H. A.; Spraul, M.; Hofmann, M.; Lindon, J. C.; Wilson, I. D.; Nicholson, J. K. 750-MHz directly coupled HPLC-NMR: Application for the sequential characterization of the positional isomers and anomers of 2-, 3-, and 4-fluorobenzoic acid glucuronides in equilibrium mixtures. *Anal. Chem.* **1995**, *67*, 4441–4445.

(15) Ogg, R. J.; Kingsley, R.; Taylor, J. S. WET, a T1-and B1-insensitive water-suppression method for in vivo localized 1H NMR spectroscopy. *J. Magn. Reson., B* **1994**, *104*, 1–10.
